# Machine learning for smell: ordinal odor strength prediction of molecular perfumery components

**DOI:** 10.1039/d6ra01805j

**Published:** 2026-05-14

**Authors:** Peter Fichtelmann, Julia Westermayr

**Affiliations:** a Wilhelm-Ostwald Institute of Physical and Theoretical Chemistry, Leipzig University Linnéstraße 2 Leipzig 04103 Germany; b Center for Scalable Data Analytics and Artificial Intelligence Dresden/Leipzig Humboldtstraße 25 Leipzig 04103 Germany julia.westermayr@uni-leipzig.de

## Abstract

Predicting olfactory perception directly from molecular structure is central to product design in a wide range of industries, such as perfumery, food and beverage, and health care. Among olfactory attributes, odor strength is a key factor in shaping odor perception, but its modeling has been impeded by scarce and fragmented intensity data. In this work, we introduce an ordinal odor strength data set of more than 2300 molecules by integrating two different public sources, mapping structures to odorless, low, medium, and high categories. Across several molecular encodings and supervised learning algorithms we compared different prediction strategies. Dimensionality reduction and SHAP analysis identified molecular shape, size and polarity as primary drivers, consistent with mass-transport constraints on volatility, sorption, and receptor access. This scalable ordinal framework enables reliable odor-strength estimation for novel molecules and provides a foundation for *in silico* fragrance design.

## Abstract 2

Predicting olfactory perception directly from molecular structure is central to product design in a wide range of industries, such as perfumery, food and beverage, and health care.

## Graphical abstract

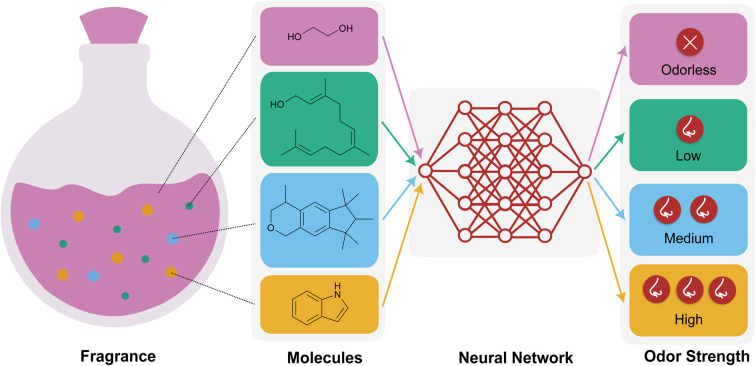

## 1 Introduction

Fragrances are ubiquitous in our daily lives. We encounter them every day in a variety of products, including perfumes, cleaning agents, hygiene articles, or care needs. However, the creation of a fragrance is a tedious process that requires fine-tuning of combinations and concentrations of up to hundreds of different raw materials. Consequently, fragrance design is costly and reserved to only a few hundred highly trained individuals worldwide with years of experience known as perfumers. For comparison, more astronauts are currently alive than perfumers.^1^

**Fig. 1:**
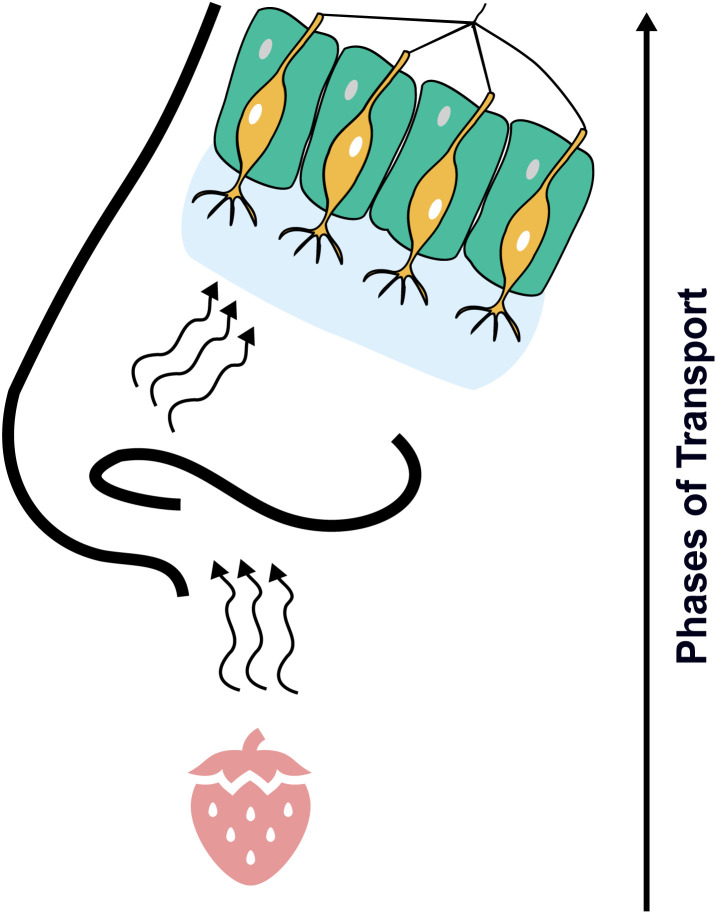
Schematic representation of the mass-transport mechanism for molecules to be olfactory stimuli. The odorant has to evaporate, enter the nose, reach the olfactory epithelium, adsorb into the olfactory mucosa, enter olfactory receptor binding pockets, and activate an olfactory receptor neuron. Thus, the chemical space of potential odorous compounds is restricted by volatility and polarity constraints.

Underlying the fragrance perception is the fact that the chemical space of odorous compounds is inherently restricted with mass transport largely determining whether a molecule is odorous or not.^2^ To be perceived, molecules must be sufficiently volatile to evaporate, travel the nose, and reach the olfactory epithelium. Yet, they also must possess the right balance of polarity and hydrophobicity to traverse the mucous layer and interact with olfactory receptors to trigger an olfactory receptor neuron. This process is illustrated in Fig. 1.

While simple rules delineate which molecules can be perceived as odorous at all,^2^ no comparable general rules with reliable predictive performance exist for more complex qualities of smell, such as odor similarity, intensity, or character, the latter often being described with words like fruity, floral, or rose. Addressing these high dimensional structure-perception relationships requires data-driven approaches, and has motivated the application of machine learning to predict odor similarity^3^ or character^4–15^ so far. For example, Lee *et al.*^4^ developed a principal odor map based on a message-passing neural network. The model was trained on odor character descriptions and generalized on odor thresholds and odor similarity. Sisson *et al.*^15^ extended this approach to binary perfumery blends. As these studies show, the predominant focus in this direction is on the character of the odor rather than its strength even though odor intensity is a decisive factor in the perception of odor. This lack of studies is further reflected in the larger data sets of thousands of compounds available containing descriptive language of odorants, such as data sets like Good Scents^16^ or Leffingwell.^17^ Only a few studies recorded odor intensity-perception data of molecules with less than 600 different investigated substances in total.^18–24^

With respect to the scarcity of intensity data, current state-of-the-art approximations are simple models to predict the psychophysical intensity curve of an individual odorant. Examples are linear (odor values),^25^ exponential (Stevens' law),^26^ or parabolic (*e.g.* Hill's model)^27^ approaches. However, such models face several limitations, such as limited accuracy for predictions at high concentrations of odorants due to the missing modeling of receptor saturation (linear and exponential) and being based on highly variable odor detection thresholds (linear and exponential).^28^ The latter does not necessarily equal perceived intensity.^29^ Recent work has begun to address this issues by predicting parabolic psychophysical curve parameters and extending these predictions to mixtures, using a set of 62 distinct molecules.^30^

**Fig. 2:**
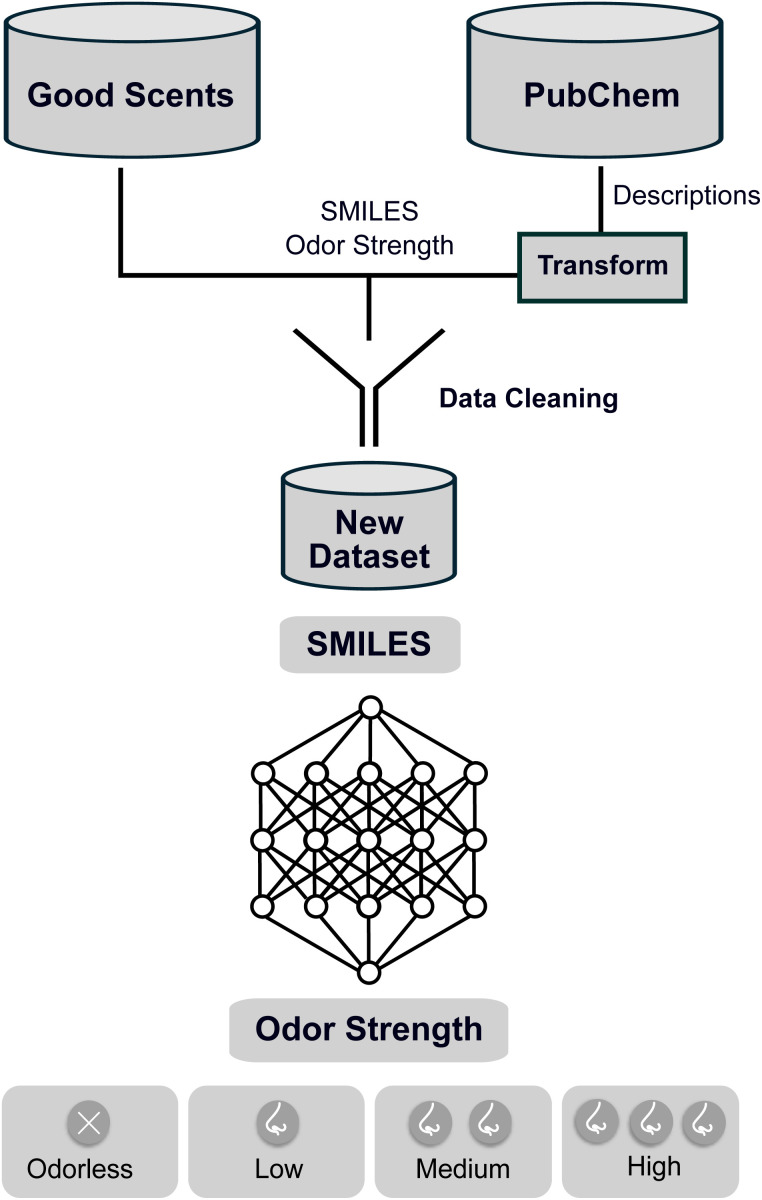
Overview of the developed method. A data set of the ordinal odor strength of more than 2000 compounds was compiled from Good Scents and PubChem. Ordinal regression with a range of state-of-the-art machine learning algorithms was performed to categorize substances by their odor strength.

To overcome the scarcity of data and allow for machine learning training on odor strength, we introduce the first odor strength data set containing over 2300 molecules that allows for generalization across a variety of odor components. Therefore, data from two different sources, namely the Good Scents Company^16^ and PubChem,^31^ were curated and combined. Using these data, we further investigated the capacity of different descriptors and regressors to predict the odor strength. To our knowledge, these are the first machine learning-based models to predict odor strength categories as an estimate of the odor intensity from molecular structures. An overview of the process is illustrated in Fig. 2.

## 2 Results and discussion

### 2.1 Curated data set and odorous chemical space

Most odor data is corporate property of fragrance houses and odor intensity data is only compiled in data sets for some hundreds of compounds.^18–24^ However, machine learning algorithms usually require large data sets for training. Therefore, the first step in our study was to curate a data set. This step is represented in Fig. 2 at the top. In particular, we evaluated the propensity to combine different sources to compile a larger data set of odor strengths than currently available. As can be seen, we cleaned and combined data from The Good Scents Company^16^ and PubChem.^31^ Data cleaning and preprocessing has to be performed as data from PubChem was labeled by intensity descriptions that had to be transformed to odor strengths. In addition, prior to merging the data, data points were removed that did not represent a valid SMILES string. Details on this process are provided in the Computational Details section.

**Fig. 3:**
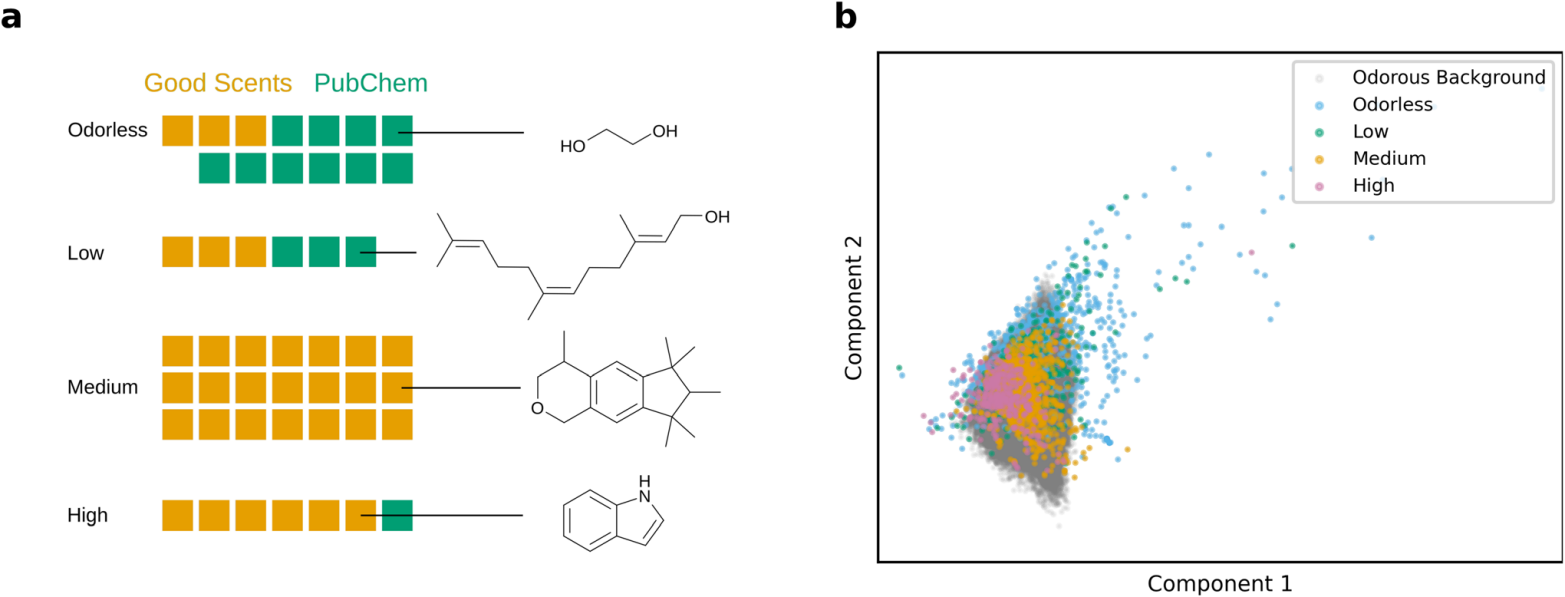
Data set representations. (a) Amount of data for each odor strength. Each square corresponds to about 50 data instances. For each odor strength category, an example molecule is shown. The table with values is shown in the SI in Table S1. (b) 2D PCA of the RDKit descriptors of our curated data set colored by their odor strength and the odorous background data set consisting of 52 457 molecules (grey) obtained from a downsample of the GDB-17 database^32^ with a predicted odor probability of 50% or more according to the best-performing model from Mayhew *et al.*^2^ Glucagon is not shown for better visability. A PCA including Glucagon is provided in Fig. S2a in the SI.

The total data set contains 2393 molecules. The odor strength distribution for each category, including representative example molecules, is illustrated in Fig. 3a. Each block in the figure corresponds to 50 data instances. The exact counts are provided in the (SI) in Table S1. Orange blocks represent data from Good Scents and green blocks indicate data from PubChem. The combination of data should balance the different categories for learning as Good Scents contains mainly medium and high odor strength molecules while PubChem contains mainly low or odorless strength components. The datasets showed a high inter-annotator agreement. We calculated two established chance-corrected metrics: (1) a quadratically weighted Cohen's kappa^33^ of 0.83 on the intersection of the Good Scents and PubChem dataset and (2) a Krippendorff's alpha^34^ of 0.81 (range 0 to 1, where 1 is perfect agreement) on the entire datasets. The later metric considers the label probability across the entire datasets and not only across the intersection. Both metrics range from 0 to 1, where 1 is perfect agreement. Consequently, we did not risk major subjective inconsistencies and our keyword-mapping approach is sufficiently reliable. Despite combining sources to improve balance, medium intensity remains the majority class and low intensity accounts for 12% of the total, which reflects the underlying availability of annotated strength labels rather than curation bias. A higher balance in odor strength is expected to increase the robustness of the trained model's performance. Additional descriptor repositories (such as those from Leffingwell^17^ or Thiboud^35^) were not merged because they provide odor character and performance notes but little to no odor strength descriptions, making harmonization across ordinal categories infeasible without unverifiable assumptions. Similarly, psychophysical intensity data sets were excluded because their ratings are explicit functions of concentration, solvent, and panel protocol; mixing them without a shared concentration scale or covariate model would confound structure-perception relationships and inject systematic bias into ordinal labels.^18–24^ This conservative choice defines a single-label, concentration-agnostic ordinal task anchored in molecular structure, while deferring integration of concentration- and solvent-explicit studies to future work where dilution and solvents can be modeled as covariates informed by established psychophysical laws of odor intensity.

Potentially one of the most critical concerns in most odor literature data is the purity of fragrance chemicals. Impurities can alter the odor even in very low concentrations.^36^ A recent study by Mayhew *et al.*^2^ using gas chromatography-olfactometry reported that 22% of the supposedly odorous molecules investigated were, in fact, odorless.^2^ We compared the 70 intersecting molecules between the GC-analyzed substance samples of the study and the compounds of our dataset. The value count of the label-label-pairs is shown in Fig. S1 in the SI. Only 2 of 57 as odorous labelled molecules by Good Scents/PubChem were odorless according to Mayhew *et al.*^2^ That are 3.5% misclassifications compared to the reported 22% odorless rate.

To characterize how curated molecules populate an odorous chemical space, the data set was embedded with principal component analysis (PCA)^37,38^ on RDKit descriptors,^39^ with projections shown in Fig. 3b. RDKit descriptors, in this case, comprised 217 structural, physicochemical, and topological parameters of molecules, such as molecular weight, octanol–water partition coefficient (log *P*), or the number of heteroatoms. PCA maps this correlated descriptor space to orthogonal principal components ranked by explained variance, enabling faithful visualization on a reduced set of axes for inspection. For context, an odorous background of 52 457 compounds was constructed by downsampling the GDB-17 database and retaining molecules with predicted odorous probability larger than 50% according to the best-performing model of Mayhew *et al.*^2^ Comparable qualitative structure is recovered when (i) excluding the odorous background, (ii) substituting UMAP (Uniform Manifold Approximation and Projection for Dimension Reduction),^40^ a non-linear dimensionality reduction method, for PCA, and (iii) swapping RDKit descriptors for circular fingerprints (Morgan bit- and count-based), with results of all alternatives reported in the SI in Fig. S2–S4. As can be seen from Fig. 3b some odorless and low-strength entries fall outside the background space, consistent with mass-transport constraints that bound olfactory space. Coverage is broad but not uniform; PubChem- and Good Scents derived entries concentrate in specific areas, reflecting the data set's deliberate emphasis on perfumery-relevant chemotypes. The PCA and UMAP representations visualized by data source are shown in the SI in Fig. S4.

Another salient pattern is that odor strength categories do not resolve into distinct clusters but instead overlap substantially in the descriptor space. This makes conventional clustering algorithms, that do not use labels but cluster data solely on input features, inaccurate for separating molecules by their odor strength. We justified this assumption by evaluating several clustering algorithms, including K-means,^41^ Gaussian mixture models,^42^ density-based spatial clustering of applications with noise (DBSCAN),^43^ spectral,^44,45^ and agglomerative^46^ clustering. No groups corresponding to odor strength were formed. Additional information can be found in the SI in Section S1.3, including evaluation metrics (adjusted rand index, normalized and adjusted mutual information) in Table S2 and the best clustering result in Fig. S7.

To analyze which features are most important for data separation, the feature importance of the principal components was analyzed. The corresponding PCA loadings indicate that the first principal component is primarily influenced by features, which describe molecular weight, size, and connectivity. The second principal component reflects contributions from heteroatoms and polarity. The exact distribution of the top 15 features to the different principal components can be found in the SI in Tables S3 and S4. These features are major factors governing mass transport of molecules from odor source to olfactory receptors. This observation aligns with findings of Mayhew *et al.*, who showed that the mass transport is key for determining whether molecules are odorous or not. Consequently, the chemical space occupied by odorous molecules becomes progressively narrower as odor strength increases, which reflects the tighter constraints imposed by volatility and polarity. Further visualization plots underlying this claim can be found in the SI in Fig. S5 and S6.

### 2.2 Odor strength predictors and model validation

As conventional cluster algorithms fail to group the data by their odor strength, further supported by dimensionality reduction and chemical space analysis in the previous section, supervised learning algorithms are used for odor strength prediction. In contrast to unsupervised learning models (clustering), supervised learning models utilize labeled data to guide the learning process. Consequently, the objective was to determine whether such a model could learn the relationship between molecular structure and odor strength of the curated data.

To predict odor strength based on molecular representations, two distinct modeling strategies were tested: First, we used a “direct approach”. For this, we defined four classes, *i.e.*, odorless, low, medium, and high odor strength, followed by training a model on all of these classes. Second, we separated odorless molecules from odorous substances. This approach then requires two predictions, one that decides whether a molecule smells or not and the second should categorize the strength of odor (low, medium, high). Both strategies were evaluated to test complementary hypotheses about the structure-to-perception pathway: a single-task ordinal learner might best capture global trade-offs across all classes, while a hierarchical, two-step pipeline could exploit different molecular features for mass transport first and for receptor interaction second.

Five regression algorithms were tested, in particular classical logistic regression,^48^ random forest,^49^ extreme gradient boosting (XGBoost),^50^ multi-layer-perceptrons (MLP),^51,52^ and consistent rank logits (CORAL),^53^ an MLP architecture designed for ordinal regression. Each algorithm was tested with seven widely used molecular encoding strategies to represent a molecule. This breadth allowed us to assess the predictive performance in fundamentally different feature spaces. We calculated structural, topological, and physicochemical descriptors (RDKit descriptors^39^) and employed classical fingerprints that highlight subtle structural differences. These included the circular substructure Morgan fingerprint,^54,55^ the predefined substructure MACCS keys fingerprint,^56^ and several topological fingerprints that capture sequences of atom connectivity for longer-range relationships, such as the RDKit fingerprint,^39^ the topological torsion fingerprint,^57^ and the atom pair fingerprint.^58^ Beyond these classical approaches, we evaluated more recent representation learning techniques. ChemBERTa-2,^59^ a language model pretrained on 77 million SMILES strings provided data-driven embeddings. Additionally, graph-based encodings using message passing neural networks have recently demonstrated promising results to predict odor character^4^ and similarity.^3^ Consequently, we applied ChemProp,^60,61^ a framework for message passing neural networks. Recognizing the potential of pretraining to potentially improve downstream model performance,^62^ we further tested CheMeleon,^63^ a foundational ChemProp model pretrained on Mordred descriptors of 77 million molecules. All model hyperparameters were optimized using 10 times repeated 10-fold cross validation. The high number of repetitions reduced the impact of noise in the data. The hyperparameters that were optimized are provided in the SI in section S2 (Tables S5–S14) for the different models used. We chose the macro averaged mean squared error (macro MSE) as the average of the MSEs computed for each odor strength categories. The equation is provided in the Computational Details section in eqn (1). The macro MSE penalizes larger errors more heavily while equally weighting all odor strength categories, regardless of how many samples they contain. In contrast, the common micro MSE is calculated globally across all classes and dominated by the majority classes. We report further metrics (micro MSE, F1 macro, F1 micro/accuracy and receiver operating characteristic area under the curve (ROC AUC, measuring a models ability to distinguish between classes across all thresholds)) on the test set for the direct approach in Table S17 and for the indirect approach in Table S18 in the SI. The prediction of the indirect model steps was combined as follows: (1) the first model predicts if the compounds are odorous or not, (2) the second model predicts the odor strength of only those compounds which were predicted as odorous in the previous step. Error metrics are computed based on the final results.

The results of the indirect approach steps are provided in the SI in Fig. S7–S11 and are generally less accurate. The difference between direct and indirect approach regarding the macro MSE values for each model and descriptor combined is plotted in Fig. S11. We performed 5 × 2 cross-validation paired *t*-tests between the direct and indirect approach for all descriptor–predictor combinations to test the significance of the performance differences. The *t*- and *p*-values are provided in Table S15. The direct approach outperformed the indirect one in 19 out of 30 descriptor–predictor combinations (confidence interval 95%). Given the reduced potential for error propagation, lower computational resource consumption during both training and prediction and generally higher performance across models and descriptors, the remainder of this study will focus on the direct approach.

**Fig. 4:**
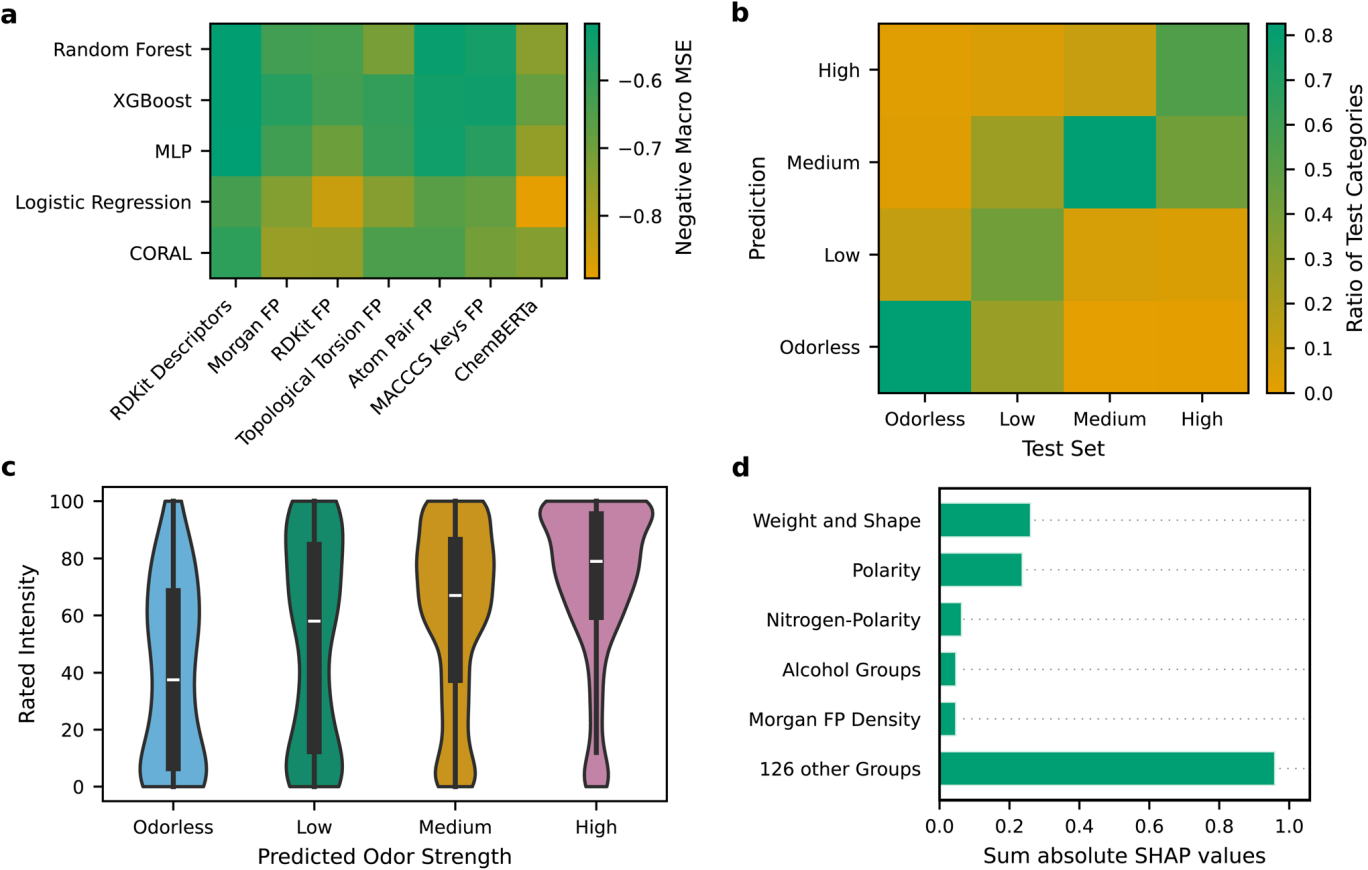
Model performance for the direct prediction approach. (a) Macro averaged mean squared error (MSE) across odor strength categories in the test set for all combinations of molecule descriptors (bottom) and predictors (left). MLP is multi-layer-perceptron and FP fingerprint. (b) Confusion matrix normed by the number of test samples for the direct ensemble model on the test set, averaged over 10 random-seeded training runs. (c) Area-normed violin plots of the direct ensemble model predictions for novel molecules from Keller *et al.*^20^ compared with their experimentally rated odor intensities (from 0 to 100; 13–108 ratings per molecule) at 10^−3^ dilution. (d) Global SHAP (SHapley Additive exPlanations)^47^ feature importance of the most influential feature groups of the direct ensemble model. The RDKit descriptor features were grouped using agglomerative clustering based on their feature value correlation (threshold: 0.75 maximizing the silhouette score (Fig. S14 in SI)). The absolute SHAP values within each group were summed.

We conducted additional 5 × 2 cross-validation paired *t*-tests between the four direct models with the lowest macro MSE in hyperparameter optimization. The results are shown in Table S16 in the SI. No significant differences were observed between these models (confidence interval 95%). Due to the better interpretability of RDKit Descriptors, we focus the rest of the study on these models. The RDKit Descriptor MLP, Random Forest and XGB models were combined into an ensemble and predictions were calculated as average of individual predictions. The normed confusion matrix of this direct ensemble model is shown in Fig. 4b. Notably, the highest accuracy was observed for the medium odor strength class, which was most prevalent in both the training and test set. In contrast, the least frequent low odor strength class showed the worst performance. Overall, most misclassifications occurred between adjacent odor strength categories. Our ensemble correctly classified 72% of the test instances within their respective categories. MSE, F1, Accuracy, and ROC AUC including class-specific scores are reported in Table S19 in the SI.

Finally, we assessed the model performance on another literature-derived test set that contains experimental odor intensity ratings.^20^ The molecules were entirely distinct from the data set derived and used above, with no close structural similarities evaluated by the Tanimoto similarity of the bit-based Morgan fingerprints. Fig. 4c displays the results using a violin plot that shows the mean of the rated intensity and its distribution among the intensity ratings (white line and black box) in addition to the distribution (shape, color) of the predictions at 10^−3^ dilution. As can be seen, predicted odor strengths correlate well with the rated odor intensities (ranging from 0 to 100). Despite considerable variability among individual ratings our model provides a reasonable approximation of perceived odor strength. This trend is further confirmed when combining dilutions ranging from 10^−3^ to 10^−7^ with a concentration difference of up to 10^4^ magnitude. The predictions of odor strength of the direct ensemble model correlate unexpectedly well even without explicit modeling of odorant concentration, as shown in Fig. S12a in the SI. However, our model is limited to lower concentrations. The ensemble model's performance degrades at a dilution level of 10^−1^ (Fig. S12b).

### 2.3 Physicochemical determinants of odor strength

While the performance of a machine learning model is a central aspect that can be assessed, for instance *via* MSEs or other metrics, model explainability is equally important.^64^ To provide insights into the factors that drive model decisions, we use SHAP (SHapley Additive exPlanations),^47^ which assigns each feature a contribution value to a model's prediction based on Shapley values^65^ from cooperative game theory. This approach provides both global and local interpretability of the direct ensemble model's behavior. To assess global feature importance, we computed the absolute SHAP values across all features and aggregated them into groups based on agglomerative clustering^46^ on their feature value correlation (threshold: 0.75 due to highest silhouette score (Fig. S14 in SI)). The resulting SHAP-based feature importance for the most influential feature groups is shown in Fig. 4d and the contributions of each feature to its corresponding group in the SI in Fig. S15–S19.

In agreement with the loadings of the first two principal components of the described PCA (Fig. 3b), features related to molecular polarity, such as the number of hydrogen acceptors or donors and the number of heteroatoms, as well as features describing molecular weight and shape, such as molecular weight or Chi descriptors,^66^ exhibit the highest importance in the direct ensemble model's predictions. Additionally, nitrogen-related polarity, the presence of alcohol groups and Morgan fingerprint density, which measures the number of non-idential subgroups, contributed substantially to the model predictions. Notably, the cumulative impact of the remaining feature groups was markedly higher, reflecting the complexity of the model's decision process. Furthermore, the feature importance per odor strength is shown in the SI in Fig. S20. No clear trends that specific properties are more relevant for higher or lower odor strengths could be observed. Representative examples of local feature group contributions for four molecules representing each of the odor strength classes are shown in the SI in Fig. S21. These examples are in line with results found globally and illustrate that polarity, molecular weight and shape substantially influence odor strength.

## 3. Conclusion

In this work, the propensity of machine learning for the prediction of odor strength was assessed. We show that odor strength can be predicted directly from molecular structure using a newly curated data set of more than 2300 odorants compiled and merged from two different sources. Two-dimensional reduced representations of the chemical odorous space showed that odor strength categories overlap significantly and do not form specific clusters when using molecular descriptors or circular fingerprints as representations, hence making unsupervised learning methods difficult. Key variation drivers to distinguish classes in odor strength were analyzed with mass-transport-related features such as molecular weight, size, shape and polarity, being more restrictive for higher odor strengths. Comprehensive benchmarking of state-of-the-art molecular encodings and predictive algorithms identified multiple algorithms as best-performing models, which showed no significant performance differences between them. We tested two different learning strategies: first, we investigated the approach of directly predicting odorless, low, medium, and high odor strength categories. Second, we examined the separation of odorless molecules and odorants first, classifying odorants by their odor strength in a second step. While both models exhibit comparable results, the first, direct approach is more stable and generally more accurate and in 19 of 30 descriptor–predictor combinations significantly better. Finally, we interpreted model feature attributions, which aligned with known principles of molecular mass transport. Our proposed model can be utilized to predict the odor strength of novel molecules and thus supports rational fragrance design, where weighting of components by odor strength could be beneficial.

One of the main challenges in the prediction of odor strengths remains the labeling of molecules, which is highly subjective and requires evaluations of many individuals for robust results. However, odor perception is highly variable between individuals.^19^ Moreover, the discrete classification itself neglects continuous variations in odor intensity within an odor strength category. To further advance odor intensity modeling, there is a need for comprehensive odor intensity data covering a wider range of molecules and mixtures, measured at multiple concentrations and with well-characterized impurities. Such data would support models that better capture the empirically known shape of the monotonic, sigmoidal relationship between the logarithm of odorant concentration and perceived odor intensity, whose slopes vary for different odorants.^67^ While odorousness^2^ and odor strength can be reliably predicted from structure-related transport properties, incorporating biological information, such as receptor responses, may yield even more accurate and mechanistically grounded models. Overall, this work provides a step towards data-driven *in silico* fragrance design.

## 4. Computational details

### 4.1. Data set generation and analysis

For data curation, a web crawler was built using the Python package BeautifulSoup^68^ to extract the odor strength, SMILES strings, and CAS-numbers of all 42234 entries on The Good Scents Company website.^16^ Due to some incorrect SMILES in the Good Scents repository, CAS-numbers were first converted into SMILES using the CAS Common Chemistry API.^69^ If this conversion turned out unsuccessful, SMILES strings from Good Scents were used. If still unavailable, CAS-numbers were converted to SMILES *via* PubChem.^31^ Since only 13 compounds were annotated with ‘very high’ odor strength, they were reclassified as ‘high’. The specific compounds are highlighted in section S6 in Table S20. Regarding the PubChem data, all compound IDs (CIDs) of entries with odor descriptions (2,393) according to the PubChem classification browser were retrieved, and corresponding SMILES and odor descriptions were obtained *via* web crawling. All SMILES strings were canonicalized using RDKit.^39^ Odor descriptions from PubChem were converted into odor strength categories if predefined keywords were present in the description. The keywords for each category are provided in the SI in section S6. To quantify the inter-annotator agreement between the Good Scents and PubChem datasets, the quadratically weighted Cohen's kappa^33^ was calculated as implemented in scikit-learn on the intersection of molecules from the dataset.^70^ In addition, Krippendorff's alpha^34^ was calculated on the entire datasets using the python library Fast Krippendorff.^71^

Only molecules with valid canonicalized SMILES were retained. Duplicated SMILES entries from PubChem were removed. A total of 332 SMILES containing dots were identified. Dots in SMILES are not part of a molecule's covalent structure, but indicate separate disconnected fragments, *e.g.* ions or isomers. The SMILES with dots and the ambiguous entries of ‘carob bean absolute’ and ‘galbanum resinoid’ were excluded. A total of 2393 data points were collected with 1678 entries from Good Scents and 715 entries from PubChem.

In addition, another independent hold-out test set was generated using data from Keller *et al.*,^20^ downloaded *via* Pyrfume.^72^ Molecules with 80% or more respective Tanimoto Similarity of their bit-based Morgan fingerprints (radius = 3, nBits = 2048) to at least one molecule in the train set were removed to ensure that the test set contained only novel compounds with proper dissimilarity to trained compounds. Four different dilution levels of compounds were available. Each molecule was rated between 13 and 108 times by independent laymen panelists providing a mean and standard deviation of rated intensities for each molecule per dilution.

### 4.2. Model training and validation

For training machine learning models, the whole data set was split into 80% training set and 20% hold-out test set as implemented in scikit-learn.^70^ The training set was used for 10-fold cross-validation over 10 random seed repetitions. The training set was split for each run into 90% training and 10% validation set to find optimal hyperparameters. The high number of repetitions was chosen to reduce chance-based variation. All splits were stratified to retain approximately the same percentage of odor strength categories in each subset. In addition, molecules with 80% or more respective Tanimoto similarity of their bit-based Morgan fingerprints (radius = 3, nBits = 2048) were grouped in the same split subsets to avoid leakage of structurally very similar compounds across sets.

We performed 5 × 2-cross validation paired *t*-tests. We adapted the corresponding code from the Python package mlxtend.^73^

The macro MSE computed across each odor strength category was used as evaluation metric, following the equation:1
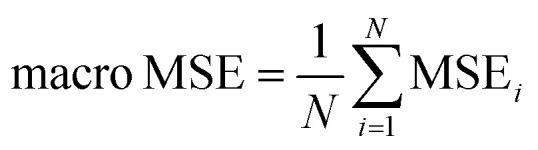
where *N* is the number of categories (four) and MSE_*i*_ the MSE for category *i*.

### 4.3. Hyperparameter optimization

Hyperparameters of each model were tuned over 100 trials with Optuna^74^ using the tree-structured Parzen estimator. In each trial, the previously described cross-validation procedure was applied. Detailed hyperparameter ranges and sampling strategies for each molecular encoder and predictor are provided in the SI in Section S2 in Tables S5–S14. Two distinct objective metrics were employed: (1) for the direct and the second step of the indirect approach: the macro-averaged MSE across odor strength categories of the validation set; (2) for the first step of the indirect approach (binary classifier: if a molecule is odorous): F1-score where the target was the minority class. To address class imbalance, all predictors used cost-sensitive learning *via* odor strength category weighted loss functions. A 25-percentile pruner discarded unpromising trials with a deviation larger than a tolerance interval (macro MSE = 0.02, F1 score = 0.015) to the best trial after each cross-validation repetition.

### 4.4. SHAP feature importance analysis

SHAP values of the test set were computed using the training set as a background to estimate average and sample feature values using the Python SHAP^47^ package. Several RDKit descriptor feature values were highly correlated (correlation matrix provided in the SI in Fig. S14). Consequently, we applied agglomerative clustering^46^ to group highly correlated features into 131 groups. A correlation threshold of 0.75 was chosen due to a maximum of the silhouette score^75^ (0.33) at this threshold. The silhouette scores for a range of thresholds are shown in Fig. S14 in the SI. While methods exist to account for feature correlations during SHAP value estimation,^76–78^ the computational cost of conditional or dependence-aware SHAP grows exponentially regarding the number of features.^79^ Regarding our number of features and number of groups, this is currently not feasible. Therefore, we report group-aggregated importance by summing SHAP values within correlation-based clusters and interpret effects at the group level.

## Author contributions

PF: data curation, machine learning training and validation, analysis, manuscript writing and revision. J. W.: conceptualization, analysis, manuscript writing and revision.

## Conflicts of interest

There are no conflicts to declare.

## Supplementary Material

RA-016-D6RA01805J-s001

## Data Availability

This study was carried out using publicly available data from the Good Scents website^16^ and PubChem.^31^ The raw Good Scents data set is protected under the U.S. and Foreign Copyright Laws. Consequently, the data set is not provided; however the code to obtain it is available. The raw PubChem data set and the code for webscrapping the raw data sets, creating and cleaning the curated data set, analyzing the chemical space, training and validating the models, revealing feature importance and applying the best-performing model with an interactive notebook to make new predictions can be found at https://github.com/peter-fichtelmann/odor-strength and is uploaded at Zenodo under DOI: https://doi.org/10.5281/zenodo.17660448 (version 1.0.7). Supplementary information (SI): further plots and tables about the curated dataset, hyperparameter ranges, model performance, model validation and SHAP feature importance analysis. See DOI: https://doi.org/10.1039/d6ra01805j.
