# Supplementary material for: Machine learning for smell: ordinal odor strength prediction of molecular perfumery components
Source: RSC Adv. 2026 May 14;16(28):25696–704. doi: 10.1039/d6ra01805j (PMC13182554; doi:10.1039/d6ra01805j)
Supplement: RA-016-D6RA01805J-s001 [file RA-016-D6RA01805J-s001.pdf]

Supplementary Information (SI) for RSC Advances.
This journal is © The Royal Society of Chemistry 2026

Supporting Information for "Machine learning for smell: Ordinal odor strength prediction of molecular perfumery components"

Peter Fichtelmann[a] and Julia Westermayr[a,b]

[a] Wilhelm-Ostwald Institute of Physical and Theoretical Chemistry, Leipzig University, Linnéstraße 2, 04103 Leipzig, Germany
[b] Center for Scalable Data Analytics and Artificial Intelligence Dresden/Leipzig, Humboldtstraße 25, 04105 Leipzig, Germany

Contents

S1 Curated data set and odorous chemical space details 2
S1.1 Dataset label distribution . . . 2
S1.2 2D representations of the odorous chemical space . . . 3
S1.3 Clustering . . . 8
S1.4 PCA loadings . . . 9

S2 Hyperparameter ranges 10
S2.1 Encoders . . . 10
S2.2 Predictors . . . 10

S3 Direct and indirect approach model performance 15

S4 Direct ensemble model validation 20

S5 SHAP feature importance analysis 21

S6 Further Method Details 28

S1 Curated data set and odorous chemical space details

Here, we additional data about the label distribution of our curated dataset, 2D representations, clustering results and PCA loadings on RDKit Descriptors.

S1.1 Dataset label distribution

The number of molecules in the curated data set regarding their odor strength categories and data set source is specified in Table S1. Regarding noise due to impurities in the compounds, we report value counts of the label-label pairs of the 70 intersecting molecules between our dataset and the GC-analyzed compounds from Mayhew *et al.* [1] in Figure S1.

Table S1 Number of molecules in the data set by odor strength. 12 Good Scents molecules with very high odor strength were counted towards high odor strength.

Odor strength	Count Good Scents	Count PubChem	Total count
odorless	144	519	663
low	156	127	283
medium	1065	0	1065
high	313	69	382
Sum	1678	715	2393

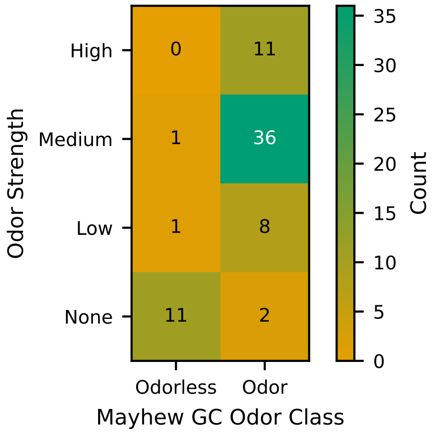

Figure S1 Value counts of the label-label pairs of the 70 intersecting molecules between our dataset and the GC-analyzed compounds in Mayhew *et al.* [1]. Only 2 of 57 as odorous labelled molecules by Good Scents/PubChem were odorless after Mayhew *et al.*. That are only 3.5% misclassifications and far from the reported 22% odorless rate.

S1.2 2D representations of the odorous chemical space

2D representations of the odorous chemical space, including molecules of our data set, are shown in Figure S2-Figure S6. We observed similar results with (Figure S2, Figure S4, Figure S5) and without (Figure S3, Figure S6) a background of potential odorous molecules (downsample from the GDB-17 database[2] with a predicted odor probability of 50% or more according to the best-performing model from Mayhew *et al.*[1]). Principal component analysis (PCA) and uniform manifold approximation and projection (UMAP) were used to reduce the dimensionality of RDKit descriptors and a bit- and count-based Morgan fingerprint(radius=3, nBits=2048) colored by odor strength in Figure S2, Figure S3, Figure S5, Figure S6 and colored by data set source in Figure S4.

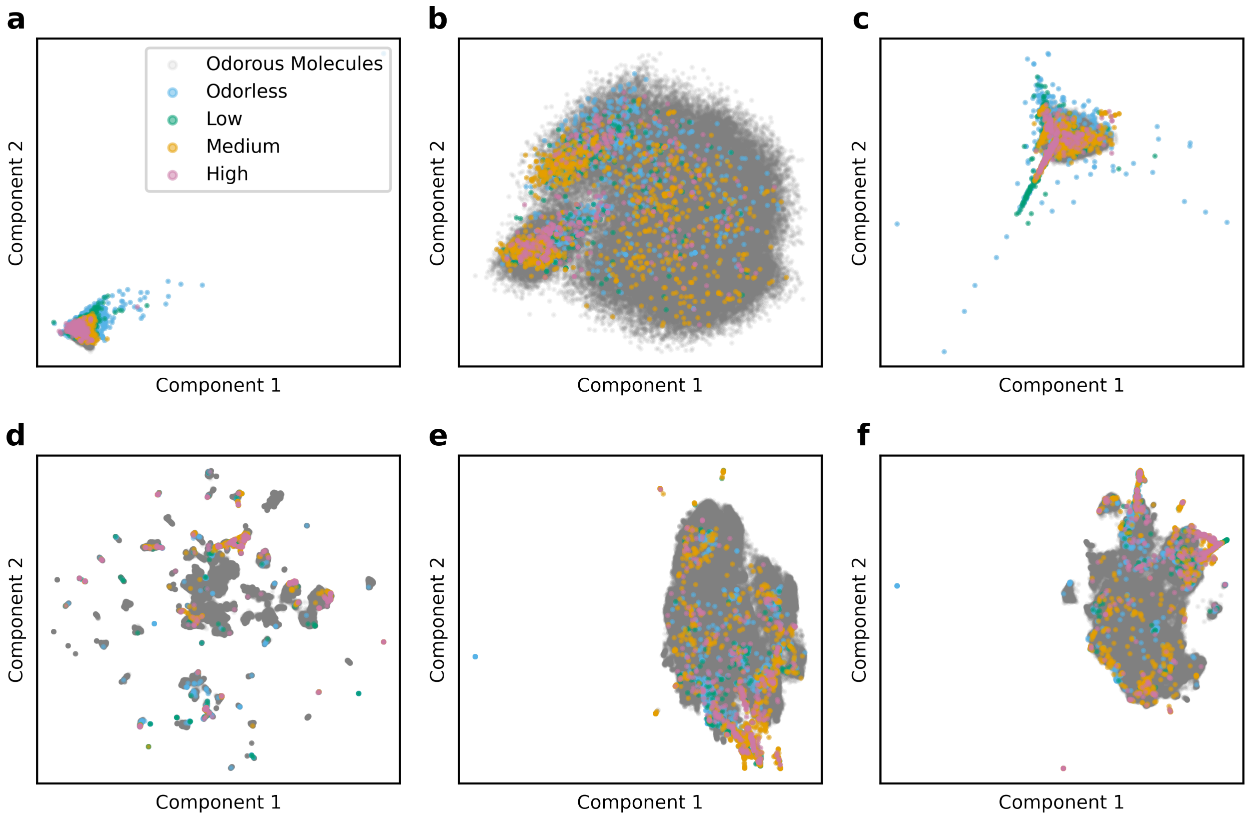

Figure S2 2D representation of our curated data set colored by their odor strength and the 52457 molecules (grey) of a downsample from the GDB-17 database[2] with a predicted odor probability of 50% or more according to the best-performing model from Mayhew *et al.*[1] using (a) PCA on RDKit Descriptors, (b) PCA on the bit-based Morgan fingerprint, (c) PCA on the count-based Morgan fingerprint, (d) UMAP on RDKit Descriptors, (e) UMAP on the bit-based Morgan fingerprint, (f) UMAP on the count-based Morgan Fingerprint. All Morgan fingerprints used a radius of 3 and a length of 2048. UMAP was performed with default hyperparameters.

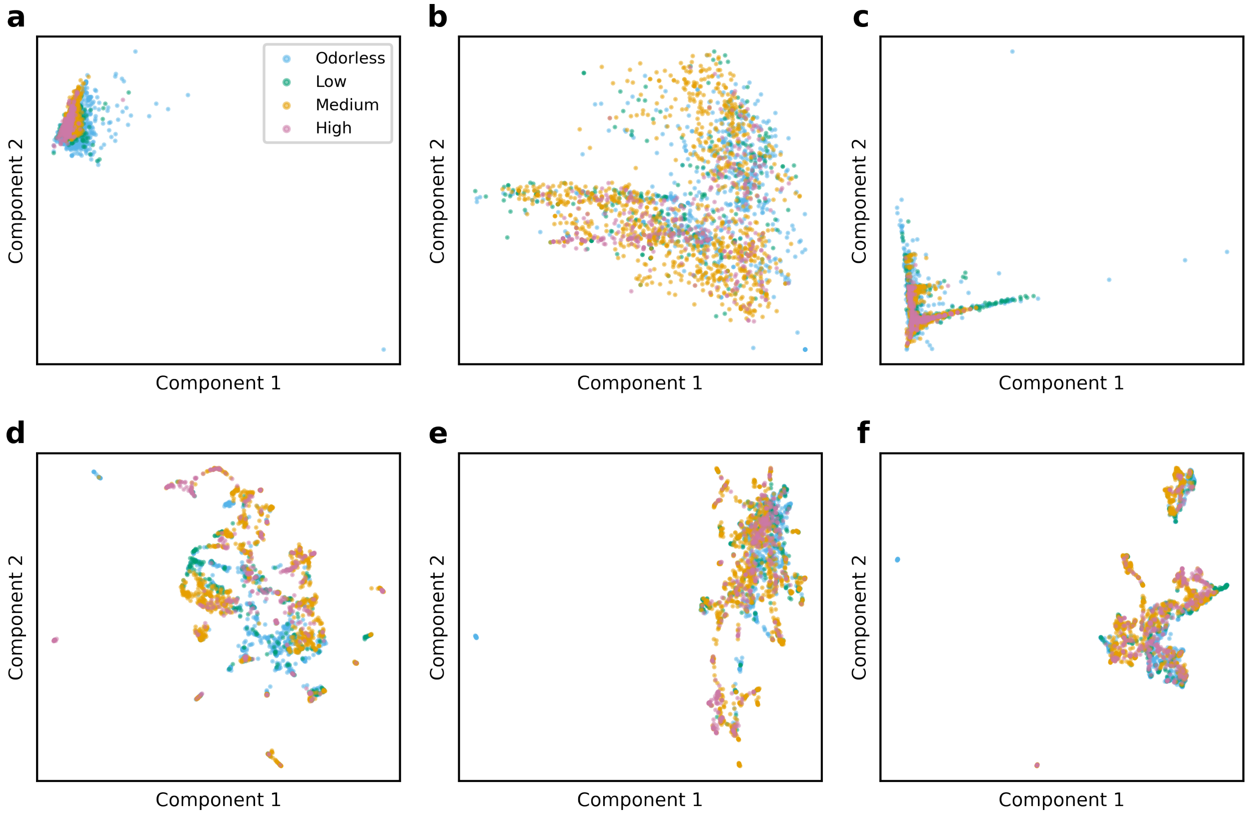

Figure S3 2D representation of our curated data set colored by their odor strength without an additional odor molecule background using (a) PCA on RDKit Descriptors, (b) PCA on the bit-based Morgan fingerprint, (c) PCA on the count-based Morgan fingerprint, (d) UMAP on RDKit Descriptors, (e) UMAP on the bit-based Morgan fingerprint, (f) UMAP on the count-based Morgan Fingerprint. All Morgan fingerprints used a radius of 3 and a length of 2048. UMAP was performed with default hyperparameters.

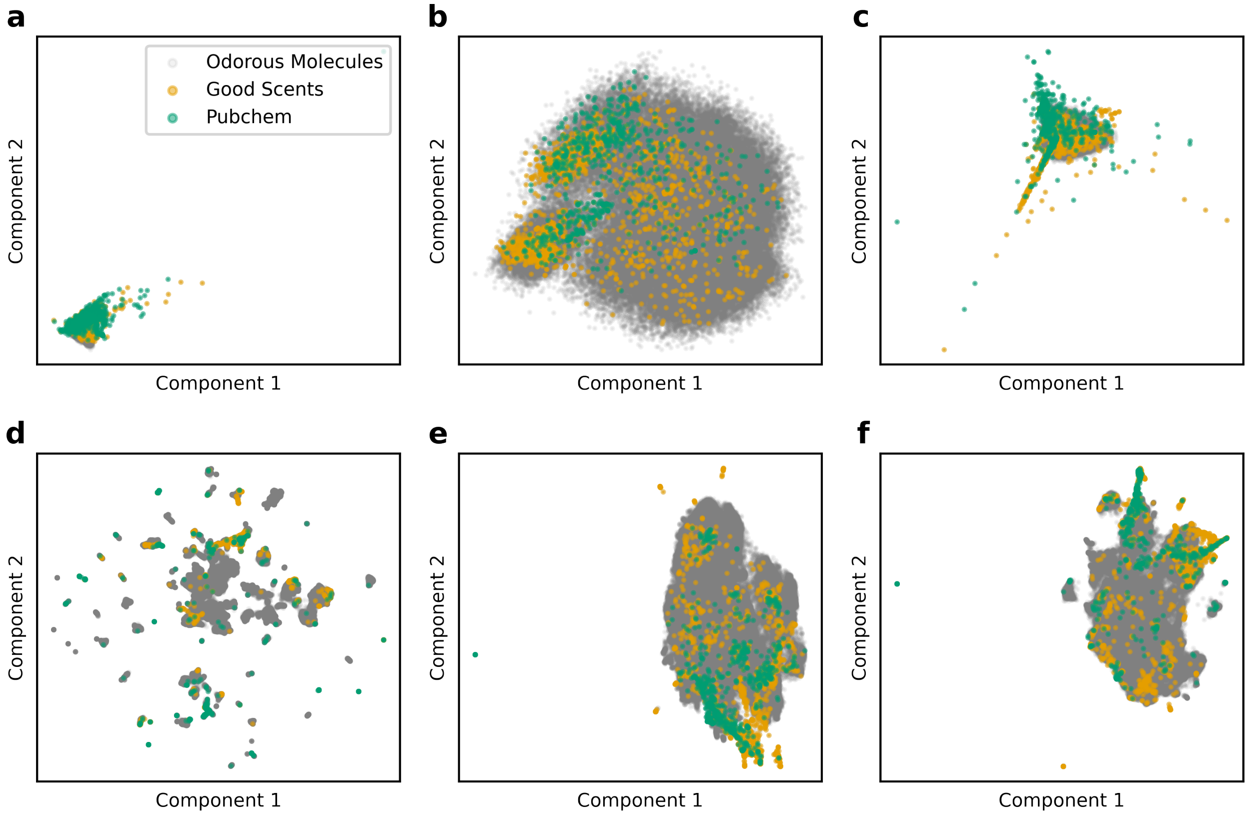

Figure S4 2D representation of our curated data set colored by their source data set and the 52457 molecules (grey) of a downsample from the GDB-17 database[2] with a predicted odor probability of 50% or more according to the best-performing model from Mayhew *et al.*[1] using (a) PCA on RDKit Descriptors, (b) PCA on the bit-based Morgan fingerprint, (c) PCA on the count-based Morgan fingerprint, (d) UMAP on RDKit Descriptors, (e) UMAP on the bit-based Morgan fingerprint, (f) UMAP on the count-based Morgan Fingerprint. All Morgan fingerprints used a radius of 3 and a length of 2048. UMAP was performed with default hyperparameters.

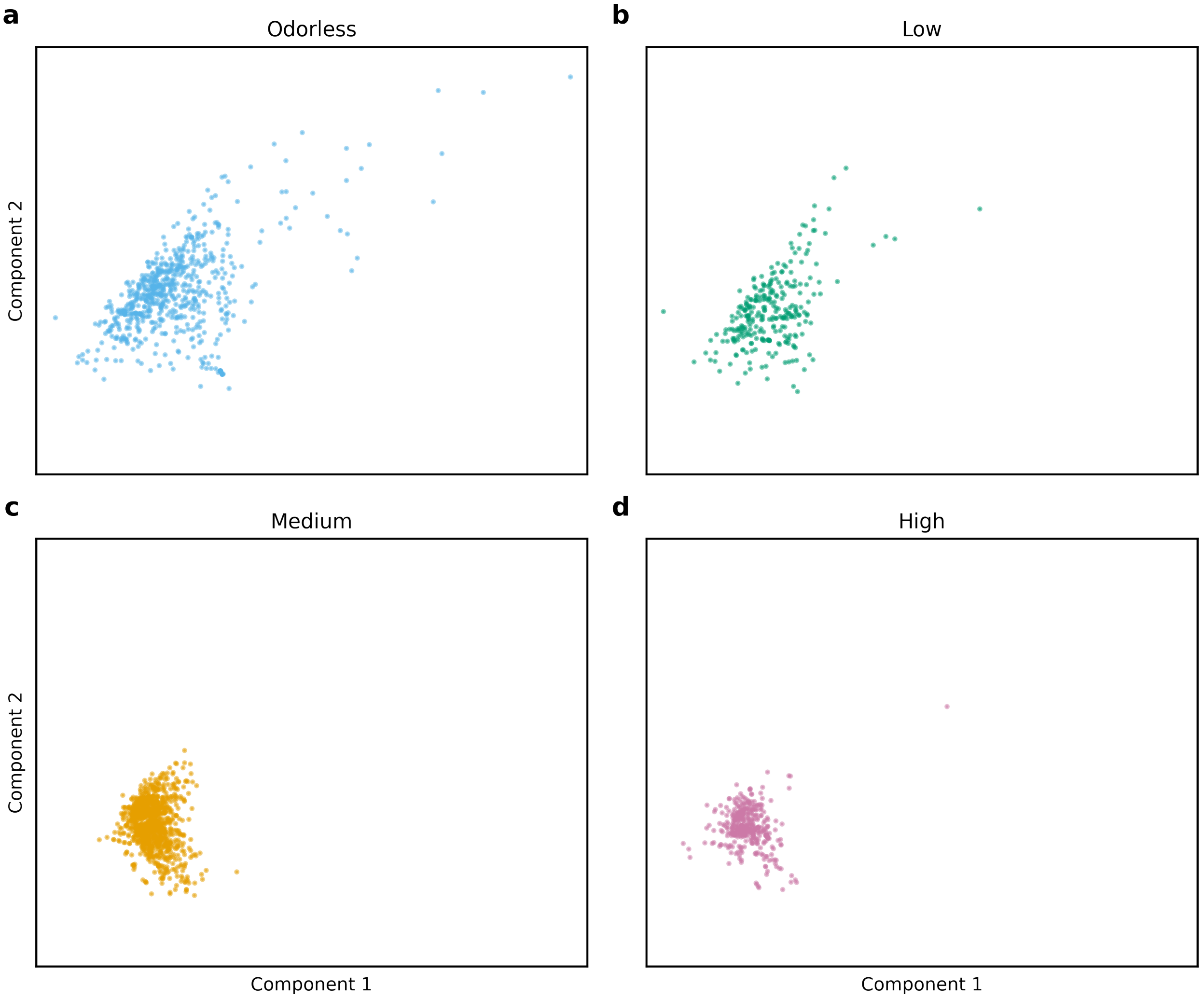

Figure S5 2D PCA of the RDKit descriptors of our curated data set and the 52457 molecules of a downsample from the GDB-17 database[2] with a predicted odor probability of 50% or more according to the best-performing model from Mayhew *et al.*[1]. (a) Odorless, (b) Low odor strength, (c) Medium odor strength, (d) High odor strength. Glucagon is not shown due to better visibility. A PCA with Glucagon is provided in Figure S2a.

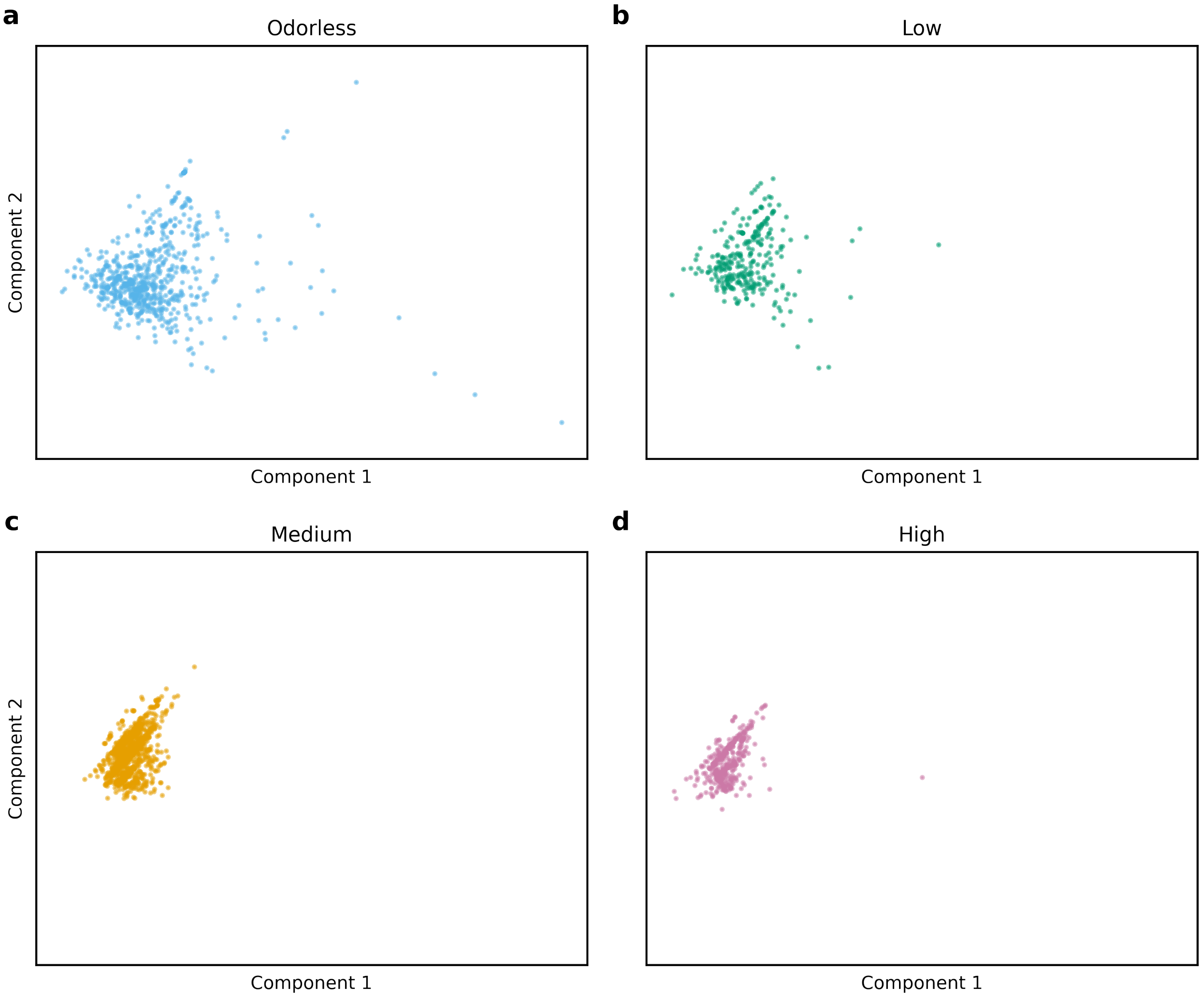

Figure S6 2D PCA of the RDKit descriptors of our curated data set. (a) Odorless, (b) Low odor strength, (c) Medium odor strength, (d) High odor strength. Glucagon is not shown due to better visibility. A PCA with Glucagon is provided in Figure S3a.

S1.3 Clustering

In Table S2 we report the adjusted rand indices (ARIs)[3], normalized mutual information (NMI)[4] and adjusted mutual information (AMI)[5] and silhouette scores[6] on our data set in comparison to the ground truth odor strengths of the investigated clustering algorithms K-means,[7] gaussian mixture models,[8] density-based spatial clustering of applications with noise (DBSCAN),[9] spectral[10,11] and agglomerative[12] clustering. For each method, we optimized key hyperparameters with respect to the adjusted rand index (ARI). The ARI is a chance-corrected measure of clustering performance compared to the ground truth ranging from 0 (random assignment) to 1 (perfect agreement). The NMI and AMI are based on mutual information and thus informational entropy. The AMI is chance-corrected while the NMI is not. Both also range from 0 (random assignment) to 1 (perfect agreement). The silhouette score corresponds to the tightness and separation of the clusters, ranging from -1 to 1 (best 1, 0 overlapping clusters). The silhouette scores reached values up to 1.0, but the ARIs, NMIs and AMIs were below 0.23. A 2D PCA RDKit Descriptors representation of the best clustering result regarding the ARI (ARI 0.23, NMI 0.17, AMI 0.17, silhouette score: 0.13) is shown in Figure S7.

Table S2 Adjusted rand index (ARI), normalized mutual information (NMI), adjusted mutual information (AMI) and silhouette scores of 5 hyper-parameter tuned clustering methods using bit-based (Morgan Binary) and count-based (Morgan Count) Morgan fingerprints (radius=3, nBits=2048) and RDKit Descriptors. The ARI. NMI and AMI were benchmarked against the odor strength categories.

Descriptor	Algorithm	ARI	NMI	AMI	Silhouette
Morgan Binary	K-Means	0.019	0.027	0.025	-0.005
Morgan Binary	GMM	0.017	0.021	0.019	-0.015
Morgan Binary	DBSCAN	0.009	0.036	0.029	1.000
Morgan Binary	Spectral	0.000	0.003	0.000	0.363
Morgan Binary	Agglomerative	0.035	0.065	0.063	0.032
Morgan Count	K-Means	0.015	0.036	0.035	0.133
Morgan Count	GMM	0.040	0.045	0.043	0.149
Morgan Count	DBSCAN	0.069	0.092	0.084	-0.047
Morgan Count	Spectral	0.000	0.003	0.001	0.595
Morgan Count	Agglomerative	0.019	0.030	0.027	0.507
RDKit Descriptors	K-Means	0.090	0.126	0.124	0.105
RDKit Descriptors	GMM	0.228	0.173	0.171	0.132
RDKit Descriptors	DBSCAN	0.004	0.034	0.027	0.649
RDKit Descriptors	Spectral	0.000	0.003	0.000	0.473
RDKit Descriptors	Agglomerative	0.148	0.155	0.153	0.160

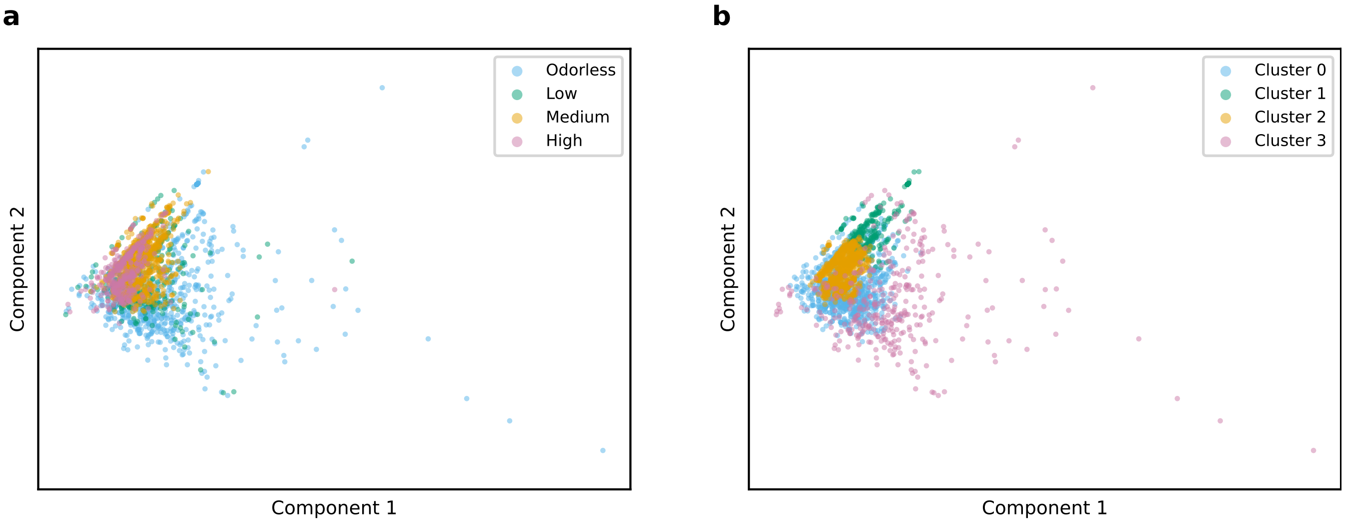

Figure S7 Best clustering result. (a) 2D PCA of the RDKit Descriptors of our curated data set colored by their odor strength. (b) 2D PCA of the RDKit Descriptors of our curated data set colored by the clusters of a gaussian mixture model, our best-performing clustering algorithm (ARI 0.23, NMI 0.17, AMI 0.17, silhouette score: 0.13). In both figures, Glucagon is not shown due to better visibility. A PCA with Glucagon is provided in Figure S3a.

S1.4 PCA loadings

The top 15 PCA loadings of the first two principal components are provided without odorous background in Table S3 and with in Table S4. In both cases, the first principal component can be attributed to molecular weight and shape and the second to heteroatoms and polarity.

Table S3 Top 15 principal components loadings (PC1, PC2) of the principal component analysis on the RDKit Descriptors of our data set.

Feature PC1	PC1 Loading	Feature PC2	PC2 Loading
Chi0	0.151237	NumAliphaticCarbocycles	0.168427
HeavyAtomCount	0.151045	SPS	0.165084
NumValenceElectrons	0.150523	NumSaturatedCarbocycles	0.157383
Chi1	0.150153	SlogP_VSA5	0.153872
LabuteASA	0.149157	VSA_EState8	0.147736
ExactMolWt	0.148014	SMR_VSA4	0.145841
MolWt	0.147922	SMR_VSA5	0.143812
HeavyAtomMolWt	0.147150	FractionCSP3	0.140981
Chi0v	0.145032	MolLogP	0.139034
Kappa1	0.144625	Chi4n	0.137100
Chi0n	0.143977	VSA_EState5	0.137054
MolMR	0.142824	BCUT2D_CHGLO	-0.133288
Chi1v	0.142211	SlogP_VSA4	0.132119
Chi1n	0.140139	VSA_EState7	0.129794
BertzCT	0.138781	SlogP_VSA1	-0.128953

Table S4 Top 15 principal components loadings (PC1, PC2) of the principal component analysis on the RDKit Descriptors of our data set and more than 50000 probable odorous molecules (from the GDB-17 database[2] downsampled molecules with a odor probability of 50% or more which was predicted with the best-performing model according to[1]).

Feature PC1	PC1 Loading	Feature PC2	PC2 Loading
Chi1v	0.170719	NumHeteroatoms	0.165858
Chi1n	0.170210	HallKierAlpha	-0.163322
NumValenceElectrons	0.169873	MinAbsPartialCharge	0.158415
HeavyAtomCount	0.169435	MaxPartialCharge	0.158190
Chi1	0.167990	NOCount	0.157602
LabuteASA	0.167783	SPS	-0.152631
Chi2v	0.167572	TPSA	0.152097
Chi2n	0.166337	SMR_VSA10	0.148329
Chi0n	0.164971	NumAliphaticRings	-0.144735
Chi0v	0.164527	NumSaturatedRings	-0.142600
ExactMolWt	0.164219	NumHAcceptors	0.138035
MolWt	0.164166	NumAliphaticCarbocycles	-0.132778
HeavyAtomMolWt	0.161262	NumSaturatedCarbocycles	-0.127086
Chi0	0.160783	FractionCSP3	-0.123140
Chi3v	0.158750	VSA_EState2	0.123060

S2 Hyperparameter ranges

Here, we provide the type, options, sampling and further conditions of the tuned hyperparameters for each molecular encoder and predictor. Hyperparameters were optimized using the tree-structured Parzen estimator (TPE) via Optuna[13].

S2.1 Encoders

The tuned hyperparameters of the Morgan fingerprint[14,15] (Table S5), the RDKit fingerprint[16] (Table S6), the topological torsion fingerprint[17] (Table S7), the atom pair fingerprint[18] (Table S8) and ChemBERTa-2[19] (Table S9) are provided. No hyperparameters of the MACCS-keys fingerprint[20] and the RDKit descriptors[16] were tuned.

Table S5 Morgan fingerprint optuna hyperparameter configuration.

Hyperparameter	Type	Options	Sampling
radius	integer	2, 3, 4	uniform
count	categorical (bool)	True, False	uniform
countSimulation	categorical (bool)	True, False	uniform
fpSize	integer	1024, 1536, 2048	uniform
includeChirality	categorical (bool)	True, False	uniform
useBondTypes	categorical (bool)	True, False	uniform
atomInvariantsGenerator	categorical	True, None	uniform

Table S6 RDKit fingerprint optuna hyperparameter configuration.

Hyperparameter	Type	Options	Sampling
count	categorical (bool)	True, False	uniform
countSimulation	categorical (bool)	True, False	uniform
fpSize	integer	1024, 1536, 2048	uniform
atomInvariantsGenerator	categorical	True, None	uniform
minPath	integer	1, 2, 3	uniform
maxPath	integer	5, 6, 7, 8, 9	uniform
useHs	categorical (bool)	True, False	uniform
branchedPaths	categorical (bool)	True, False	uniform
useBondOrder	categorical (bool)	True, False	uniform
numBitsPerFeature	integer	1, 2, 3	uniform

Table S7 Topological torsion fingerprint optuna hyperparameter configuration.

Hyperparameter	Type	Options	Sampling
count	categorical (bool)	True, False	uniform
countSimulation	categorical (bool)	True, False	uniform
fpSize	integer	1024, 1536, 2048	uniform
includeChirality	categorical (bool)	True, False	uniform
torsionAtomCount	integer	2, 3, 4, 5, 6	uniform

S2.2 Predictors

The tuned hyperparameters of the predictors logistic regression[21] (Table S10), random forest[22] (Table S11), extreme gradient boosting (XGBoost)[23] (Table S12), multi-layer-perceptron (MLP)[24,25] and consistent rank logits (CORAL)[26] (Table S13) are provided. In addition, the message-passing neural network framework ChemProp[27,28] both with and without initialization from the foundational model CheMeleon[29] was optimized. The tested hyperparameters are in Table S14. No hyperparameters of the average prediction were tuned

Table S8 Atom pair fingerprint optuna hyperparameter configuration.

Hyperparameter	Type	Options	Sampling
count	categorical (bool)	True, False	uniform
countSimulation	categorical (bool)	True, False	uniform
minDistance	integer	1, 2, 3	uniform
maxDistance	integer	5, 6, 7, 8, 9	uniform
includeChirality	categorical (bool)	True, False	uniform
fpSize	integer	1024, 1536, 2048	uniform
atomInvariantsGenerator	categorical	True, None	uniform

Table S9 ChemBERTa optuna hyperparameter configuration.

Hyperparameter	Type	Options	Sampling
target_layer	integer	0, 1, 2, 3, 4, 5, 6	uniform
pooling	categorical	'mean', 'cls'	uniform

Table S10 Logistic regression optuna hyperparameter configuration.

Hyperparameter	Type	Options	Sampling	Condition
standardizer_name	categorical	'standard', 'robust', 'minmax', 'yeo-johnson', None	uniform	—
penalty	categorical	'l2', 'l1', 'elasticnet', None	uniform	—
tol	float	1e-5-1e-3	log-uniform	—
C	float	1e-2-1e2	log-uniform	defined if penalty not None
l1_ratio	float	0.1-0.9	uniform (step size 0.1)	defined if penalty = 'elasticnet'
solver	categorical	'liblinear', 'saga'	uniform	if penalty = 'l1'
solver	categorical	'saga', 'newton-cg', 'lbfgs'	uniform	if penalty = None
solver	categorical	'liblinear', 'saga', 'newton-cg', 'lbfgs'	uniform	if penalty = 'l2'
binarize_labels	categorical (bool)	True, False	uniform	if labels ordinal (not only binary)
dealing_with_incosistency	categorical	'sum', 'max'	uniform	defined if binarize_labels = True

Table S11 Random forest optuna hyperparameter configuration.

Hyperparameter	Type	Options	Sampling	Condition
objective	categorical	'classification', 'regression'	uniform	—
n_estimators	integer	50-500	uniform (step size 50)	—
max_depth	integer	5-20	uniform (step size 5)	—
min_samples_split	integer	2, 3, 4	uniform	—
min_samples_leaf	integer	1, 3, 5	uniform	—
min_weight_fraction_leaf	float	0.0, 0.2, 0.4	uniform	—
max_features	categorical	'sqrt', 'log2', None	uniform	—
max_leaf_nodes	integer	25, 50, 75, 100	uniform	—
min_impurity_decrease	float	0.0, 0.05, 0.1	uniform	—
criterion	categorical	'gini', 'entropy', 'log_loss'	uniform	if objective = 'classification'
binarize_labels	categorical (bool)	True, False	uniform	if objective = 'classification' and labels ordinary (not only binary)
dealing_with_incosistency	categorical	'sum', 'max'	uniform	if binarize_labels = True

Table S12 XGBoost Hyperparameter Space with Conditional Dependencies

Hyperparameter	Type	Options	Sampling	Condition
objective	categorical	'binary:logistic', 'multi:softmax', 'reg:squarederror'	uniform	only binary labels: always 'binary:logistic'
custom_metric	fixed	'mse_macro' or 'f1_score'	—	only binary labels: 'f1_score'; ordinary labels: 'mse_macro'
booster	categorical	'gbtree', 'dart'	uniform	—
num_boost_round	fixed	250	—	—
early_stopping_rounds	fixed	50	—	—
eta	float	0.005-0.5	log-uniform	—
lambda	float	0.001-10	log-uniform	—
alpha	float	0.001-10	log-uniform	—
gamma	float	0.001-10	log-uniform	—
max_depth	integer	4, 6, 8, 10	uniform	—
min_child_weight	float	0.01-100	log-uniform	—
max_delta_step	float	0, 1, 2	uniform	—
subsample	float	0.6, 0.8, 1.0	uniform	—
colsample_bytree	float	0.6, 0.8, 1.0	uniform	—
colsample_bylevel	float	0.6, 0.8, 1.0	uniform	—
num_parallel_tree	integer	1, 2, 3	uniform	—
rate_drop	float	0.0, 0.2, 0.4	uniform	booster = 'dart'
skip_drop	float	0.0, 0.2, 0.4	uniform	booster = 'dart'
normalize_type	categorical	'tree', 'forest'	uniform	booster = 'dart'
sample_type	categorical	'uniform', 'weighted'	uniform	booster = 'dart'
binarize_labels	categorical (bool)	True, False	uniform	if labels ordinal (not only binary)
dealing_with_incosistency	categorical	'sum', 'max'	uniform	if binarize_labels = True
multi_strategy	categorical	'one_output_per_tree', 'multi_output_tree'	uniform	if binarize_labels = True and booster = 'gbtree'
num_class	fixed	4	—	if objective = 'multi:softmax'

Table S13 MLP and CORAL predictor optuna hyperparameter configuration.

Hyperparameter	Type	Options	Sampling	Condition
standardizer_name	categorical	'standard', 'robust', 'minmax', 'yeo-johnson', None	uniform	—
n_layers	integer	2-12	uniform (step size 2)	—
dim	integer	32-512	log-uniform	—
batch_size	categorical	16, 32, 64, 128, 256	uniform	—
n_epochs	fixed	250	—	—
early_stopping_rounds	fixed	50	—	—
delta	fixed	0	—	—
objective_name	categorical	'binary_crossentropy', 'mse', 'coral'	uniform	'coral' only for CORAL predictor (fixed); if labels binary only 'binary_crossentropy'
learning_rate	float	5e-6-5e-4	log-uniform	—
optimizer_name	categorical	'adam', 'adamw', 'sgd', 'rmsprop', 'adagrad'	uniform	—
training_scheduler_name	categorical	None, 'ReduceLROnPlateau', 'CosineAnnealingLR', 'CosineAnnealingWarmRestarts'	uniform	—
warmup_epochs	integer	0, 10, 20, 30	uniform	—
activation	categorical	'relu', 'leaky-relu', 'tanh', 'sigmoid', 'elu'	uniform	—
dropout	float	0.0-0.5	uniform (step size 0.1)	—
weight_average	categorical	False, 'swa', 'ema'	uniform	—
metric_name	fixed	'mse_macro' or 'f1_score'	—	binary labels 'f1_score'; ordinary labels: 'mse_macro'
weight_decay	float	1e-5-1e-3	log-uniform	—
betas	tuple(float, float)	beta1: 0.85, 0.90, 0.95, beta2: 0.990, 0.993, 0.996, 0.999	uniform	if optimizer in ['adam', 'adamw']
momentum	float	0.0-0.5	uniform (step size 0.1)	if optimizer in ['sgd', 'rmsprop']
nesterov	categorical	True, False	uniform	if optimizer = 'sgd' and momentum > 0
dampening	float	0.0, 0.05, 0.1	uniform	if optimizer = 'sgd' and nesterov = False
alpha	float	0.9-0.99	uniform (step size 0.03)	if optimizer = 'rmsprop'
lr_decay	float	0.0, 0.05, 0.1	uniform	if optimizer = 'adagrad'
factor	float	0.1-0.5]	uniform (step size 0.1)	if scheduler = 'ReduceLROnPlateau'
T_0	integer	10, 20, 30, 40	uniform	if scheduler = 'CosineAnnealingWarmRestarts'
T_mult	integer	2, 3, 4	uniform	if scheduler = 'CosineAnnealingWarmRestarts'
binarize_labels	categorical (bool)	True, False	uniform	if labels ordinal (not only binary)
dealing_with_incosistency	categorical	'sum', 'max'	uniform	if binarize_labels = True

Table S14 ChemProp and CheMeleon MPNN predictor optuna hyperparameter configuration.

Hyperparameter	Type	Options	Sampling	Condition
epochs	fixed	250 ChemProp, 100 CheMeleon	—	—
early_stopping_rounds	fixed	50 ChemProp, 25 CheMeleon	—	—
delta	fixed	0	—	—
mode	fixed	'min'	—	—
batch_size	categorical	16, 32, 64, 128, 256	uniform	—
aggregation	categorical	'norm', 'sum', 'mean', 'attentive'	uniform	—
n_layers	integer	1–12	uniform	—
objective	categorical	'binary_crossentropy', 'mse'	uniform	if labels binary only 'binary_crossentropy'
predictor_dim	integer	32–512	log-uniform	—
predictor_dropout	float	0.0–0.5	uniform (step size 0.1)	—
predictor_activation	categorical	'relu', 'leakyrelu', 'prelu', 'tanh', 'elu'	uniform	—
warmup_epochs	integer	0, 10, 20, 30	uniform	—
initial_learning_rate	float	5e-6–1e-3	log-uniform	—
max_learning_rate_factor	float	1–100	log-uniform	used to compute max_learning_rate
final_learning_rate_factor	float	0.01–10	log-uniform	used to compute final_learning_rate
max_learning_rate	derived	—	—	initial_learning_rate × max_learning_rate_factor
final_learning_rate	derived	—	—	initial_learning_rate × final_learning_rate_factor
message_passing	categorical	'bond', 'atom', 'chemeleon'	uniform	'chemeleon' only for CheMeleon predictor (fixed)
mp_dim	integer	32–512	log-uniform	only ChemProp predictor
bias	categorical (bool)	True, False	uniform	only ChemProp predictor
n_message_passing_iterations	integer	1–8	uniform	only ChemProp predictor
messages_undirected_edges	categorical (bool)	True, False	uniform	only ChemProp predictor
mp_dropout	float	0.0–0.5	uniform (step size 0.1)	only ChemProp predictor
mp_activation	categorical	'relu', 'leakyrelu', 'prelu', 'tanh', 'elu'	uniform	only ChemProp predictor
binarize_labels	categorical (bool)	True, False	uniform	if labels ordinal (not only binary)
dealing_with_incosistency	categorical	'sum', 'max'	uniform	if binarize_labels = True

S3 Direct and indirect approach model performance

Results of odor strength prediction using the indirect approach, consisting of two models to predict first whether a molecule is odorous and if so to predict its odor strength, are further shown in this section. The results of 5x2 cross-validation paired t-tests between the direct and indirect models of the same descriptor-predictor combinations are reported in Table S15. The direct approach was significant better in 19 of 30 cases (confidence interval 95%). Further 5x2 cross-validation paired t-tests between the 5 models with lowest macro MSE in hyperparameter optimization of the direct approach were performed. The result are shown in Table S16. Four models showed no significant differences in performance (confidence interval 95%).

The validation results on the test set are provided in Table S17 for the direct and in Table S18 for the indirect approach. The metrics including further class-specific metrics of the direct ensemble model (RDKit Descriptors with MLP, Random Forest, XGB) are reported in Table S19.

Figure S8, Figure S9 and Figure S10 provide a comparison of the first, second and combined steps of the indirect approach, respectively, across the tested algorithms for molecular encoding and odor prediction. The macro MSE difference to the direct approach is shown in Figure S11.

Table S15 5x2-Cross validation paired t-test results between the same descriptor-predictor combindations of the direct and combined indirect approach. A p-value below 0.05 is rejecting the null hypothesis that both models perform equally well with a confidence interval of 95%.

Descriptor	Predictor	t value	p value
None	ChemProp	-2.05	0.10
None	CheMeleon	-4.11	0.01
RDKit Descriptors	Random Forest	-2.38	0.06
RDKit Descriptors	XGBoost	-5.32	0.00
RDKit Descriptors	MLP	-1.40	0.22
RDKit Descriptors	Logistic Regression	-2.52	0.05
Morgan FP	Random Forest	-2.39	0.06
Morgan FP	XGBoost	-10.70	0.00
Morgan FP	MLP	-48.08	0.00
Morgan FP	Logistic Regression	-2.10	0.09
RDKit FP	Random Forest	-8.45	0.00
RDKit FP	XGBoost	-4.69	0.01
RDKit FP	MLP	0.97	0.38
RDKit FP	Logistic Regression	-22.47	0.00
Topological Torsion FP	Random Forest	-6.38	0.00
Topological Torsion FP	XGBoost	-18.03	0.00
Topological Torsion FP	MLP	-1.27	0.26
Topological Torsion FP	Logistic Regression	-6.56	0.00
Atom Pair FP	Random Forest	-1.95	0.11
Atom Pair FP	XGBoost	-1.90	0.12
Atom Pair FP	MLP	-3.04	0.03
Atom Pair FP	Logistic Regression	-2.61	0.05
MACCCS Keys FP	Random Forest	-7.54	0.00
MACCCS Keys FP	XGBoost	-7.72	0.00
MACCCS Keys FP	MLP	-3.20	0.02
MACCCS Keys FP	Logistic Regression	-3.02	0.03
ChemBERTa	Random Forest	-1.94	0.11
ChemBERTa	XGBoost	-3.37	0.02
ChemBERTa	MLP	-3.32	0.02
ChemBERTa	Logistic Regression	-0.10	0.93

Table S16 5x2-Cross validation paired t-test p-values between the 4 models with the lowest macro MSE in hyperparameter optimization of the direct approach. A p-value below 0.05 is rejecting the null hypothesis that both models perform equally well with a confidence interval of 95%.

	RDKit Descriptors | Random Forest	RDKit Descriptors | MLP	RDKit Descriptors | XGBoost	None | CheMeleon
RDKit Descriptors | Random Forest	–	0.95	0.89	0.73
RDKit Descriptors | MLP	0.95	–	0.96	0.55
RDKit Descriptors | XGBoost	0.89	0.96	–	0.75
None | CheMeleon	0.73	0.55	0.75	–

Table S17 Performance metrics of the descriptor-predictor combinations of the direct approach on the test set. Note that under this multi-class conditions the F1 score is identical to classification accuracy. The ROC AUC is calculated as sum of multiple binary problems in an ordinal one-vs-rest method.[?]

Descriptor	Predictor	MSE macro	MSE micro	F1 macro	Accuracy/F1 micro	ROC AUC
Atom Pair FP	CORAL	0.64	0.51	0.62	0.70	0.86
Atom Pair FP	Logistic Regression	0.66	0.59	0.58	0.61	0.85
Atom Pair FP	MLP	0.54	0.45	0.64	0.70	0.89
Atom Pair FP	Random Forest	0.53	0.42	0.54	0.62	0.90
Atom Pair FP	XGBoost	0.55	0.43	0.63	0.69	0.88
ChemBERTa	CORAL	0.73	0.59	0.52	0.65	0.82
ChemBERTa	Logistic Regression	0.89	0.73	0.49	0.55	0.79
ChemBERTa	MLP	0.76	0.55	0.43	0.60	0.85
ChemBERTa	Random Forest	0.74	0.60	0.52	0.61	0.81
ChemBERTa	XGBoost	0.69	0.53	0.51	0.58	0.85
MACCCS Keys FP	CORAL	0.71	0.57	0.59	0.67	0.85
MACCCS Keys FP	Logistic Regression	0.68	0.55	0.58	0.64	0.84
MACCCS Keys FP	MLP	0.58	0.43	0.38	0.62	0.90
MACCCS Keys FP	Random Forest	0.55	0.41	0.58	0.64	0.90
MACCCS Keys FP	XGBoost	0.54	0.46	0.65	0.70	0.88
Morgan FP	CORAL	0.77	0.63	0.56	0.65	0.83
Morgan FP	Logistic Regression	0.73	0.60	0.50	0.59	0.81
Morgan FP	MLP	0.62	0.47	0.62	0.66	0.88
Morgan FP	Random Forest	0.62	0.51	0.48	0.59	0.84
Morgan FP	XGBoost	0.58	0.42	0.46	0.62	0.90
None	ChemProp	0.58	0.51	0.61	0.64	0.88
None	CheMeleon	0.53	0.36	0.50	0.69	0.91
RDKit Descriptors	CORAL	0.59	0.48	0.61	0.70	0.87
RDKit Descriptors	Logistic Regression	0.63	0.52	0.57	0.65	0.84
RDKit Descriptors	MLP	0.51	0.44	0.66	0.71	0.89
RDKit Descriptors	Random Forest	0.52	0.39	0.63	0.72	0.88
RDKit Descriptors	XGBoost	0.52	0.40	0.64	0.71	0.89
RDKit FP	CORAL	0.76	0.61	0.59	0.67	0.84
RDKit FP	Logistic Regression	0.84	0.73	0.56	0.61	0.81
RDKit FP	MLP	0.69	0.61	0.62	0.66	0.85
RDKit FP	Random Forest	0.63	0.52	0.58	0.64	0.86
RDKit FP	XGBoost	0.63	0.52	0.65	0.72	0.86
Topological Torsion FP	CORAL	0.64	0.51	0.57	0.68	0.87
Topological Torsion FP	Logistic Regression	0.74	0.65	0.57	0.61	0.84
Topological Torsion FP	MLP	0.61	0.54	0.61	0.64	0.88
Topological Torsion FP	Random Forest	0.71	0.56	0.48	0.57	0.86
Topological Torsion FP	XGBoost	0.60	0.52	0.62	0.67	0.86

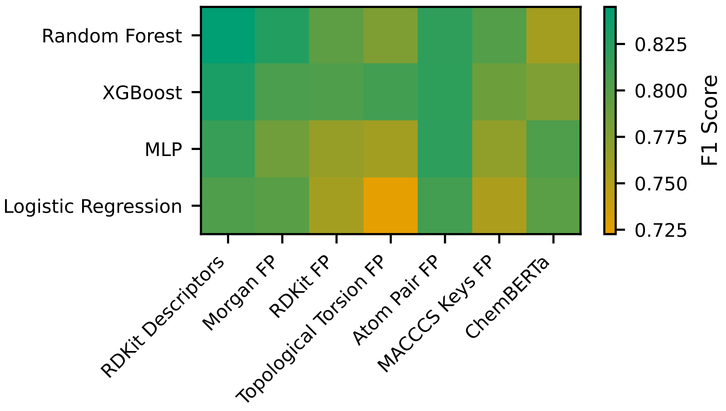

Figure S8 Model performance first step of the indirect approach (binary classifier). F1-score (target minority class) of the hold-out test set of all combinations of molecule encoder (bottom) and predictor (left). MLP is multi-layer-perceptron and FP fingerprint. The message passing neural network ChemProp[27,28] achieved an F1-score of 0.81, and the foundational model CheMeleon[29] of 0.83.

Table S18 Performance metrics of the descriptor-predictor combinations of the combined indirect approach (identical descriptor-predictor combination in both steps) on the test set. Note that under this multi-class conditions the F1 score is identical to classification accuracy. The ROC AUC is calculated as sum of multiple binary problems in an ordinal one-vs-rest method.[?]

Descriptor	Predictor	MSE macro	MSE micro	F1 macro	Accuracy/F1 micro	ROC AUC
Atom Pair FP	Logistic Regression	0.67	0.56	0.65	0.71	0.87
Atom Pair FP	MLP	0.59	0.41	0.63	0.69	0.91
Atom Pair FP	Random Forest	0.71	0.48	0.57	0.70	0.88
Atom Pair FP	XGBoost	0.68	0.44	0.58	0.67	0.89
ChemBERTa	Logistic Regression	0.93	0.77	0.55	0.61	0.83
ChemBERTa	MLP	0.85	0.61	0.53	0.58	0.86
ChemBERTa	Random Forest	0.85	0.64	0.48	0.64	0.84
ChemBERTa	XGBoost	0.83	0.58	0.51	0.63	0.85
MACCCS Keys FP	Logistic Regression	0.81	0.67	0.56	0.66	0.86
MACCCS Keys FP	MLP	0.75	0.63	0.56	0.55	0.85
MACCCS Keys FP	Random Forest	0.73	0.60	0.61	0.69	0.86
MACCCS Keys FP	XGBoost	0.68	0.47	0.57	0.67	0.89
Morgan FP	Logistic Regression	0.87	0.69	0.59	0.68	0.84
Morgan FP	MLP	0.77	0.56	0.57	0.67	0.87
Morgan FP	Random Forest	0.71	0.56	0.63	0.74	0.86
Morgan FP	XGBoost	0.59	0.40	0.57	0.69	0.90
None	ChemProp	0.71	0.46	0.57	0.66	0.89
None	CheMeleon	0.62	0.39	0.63	0.73	0.91
RDKit Descriptors	Logistic Regression	0.72	0.61	0.62	0.69	0.86
RDKit Descriptors	MLP	0.58	0.42	0.61	0.68	0.89
RDKit Descriptors	Random Forest	0.58	0.39	0.60	0.70	0.90
RDKit Descriptors	XGBoost	0.59	0.37	0.60	0.69	0.91
RDKit FP	Logistic Regression	0.99	0.85	0.61	0.66	0.81
RDKit FP	MLP	0.78	0.57	0.57	0.64	0.87
RDKit FP	Random Forest	0.87	0.60	0.55	0.69	0.85
RDKit FP	XGBoost	0.67	0.45	0.57	0.68	0.89
Topological Torsion FP	Logistic Regression	0.88	0.79	0.57	0.62	0.84
Topological Torsion FP	MLP	0.80	0.59	0.53	0.58	0.87
Topological Torsion FP	Random Forest	0.80	0.61	0.51	0.65	0.85
Topological Torsion FP	XGBoost	0.62	0.41	0.56	0.69	0.89

Table S19 Performance metrics of the ensemble model of the direct approach (RDKit Descriptors with MLP, Random Forest, XGB) including class specific metrics. Note that under this multi-class conditions the F1 score is identical to classification accuracy. The ROC AUC is calculated as sum of multiple binary problems in an ordinal one-vs-rest method.[?] We report the thresholds (low: larger equal low, medium: larger equal medium, high: larger equal high).

Metric	Metric value
MSE Macro	0.49
MSE Odorless	0.44
MSE Low	0.72
MSE Medium	0.17
MSE High	0.64
MSE Micro	0.35
F1 Macro	0.66
F1 Odorless	0.83
F1 Low	0.44
F1 Medium	0.79
F1 High	0.57
Accuracy/F1 Micro	0.72
ROC AUC	0.91
ROC AUC Low Threshold	0.94
ROC AUC Medium Threshold	0.94
ROC AUC High Threshold	0.85

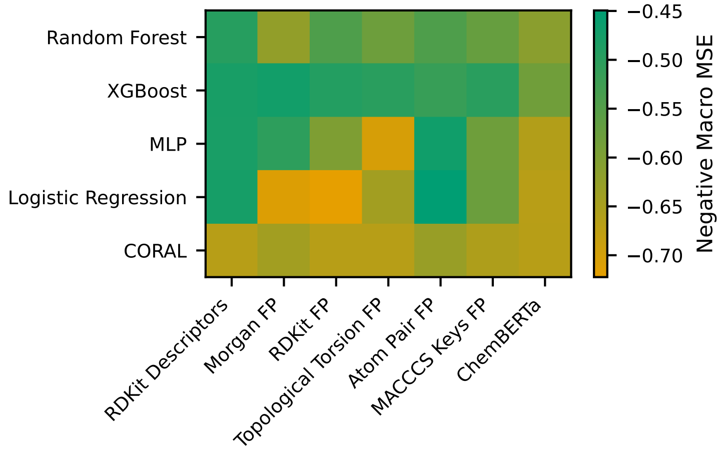

Figure S9 Model performance second step of the indirect approach. Macro averaged mean squared error (MSE) over the categories of the hold-out test set of all combinations of molecule encoder (bottom) and predictor (left). MLP is multi-layer-perceptron and FP fingerprint. The message passing neural network using ChemProp[27,28] achieved a score of 0.52, and the foundational model CheMeleon[29] of 0.43.

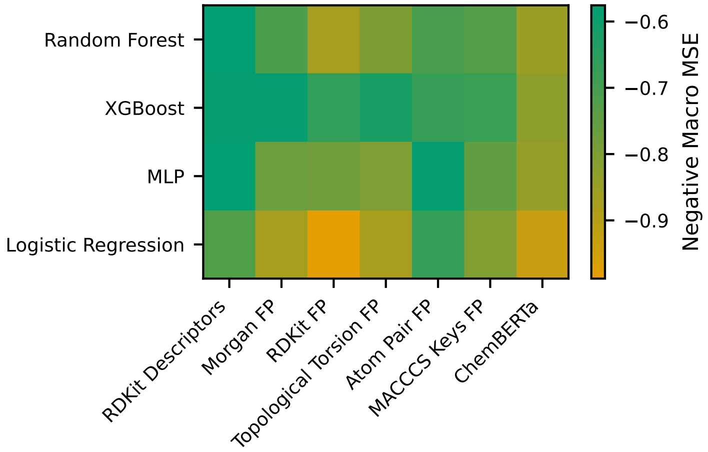

Figure S10 Model performance combined steps of the indirect approach. Macro averaged mean squared error (MSE) over the categories of the hold-out test set of the cross-validation for the best hyperparameter optimized models of the same model combinations between the steps (molecule encoder (bottom) and predictor (left)). MLP is multi-layer-perceptron and FP fingerprint. The message passing neural network using ChemProp[27,28] achieved a score of 0.71, and the foundational model CheMeleon[29] of 0.62.

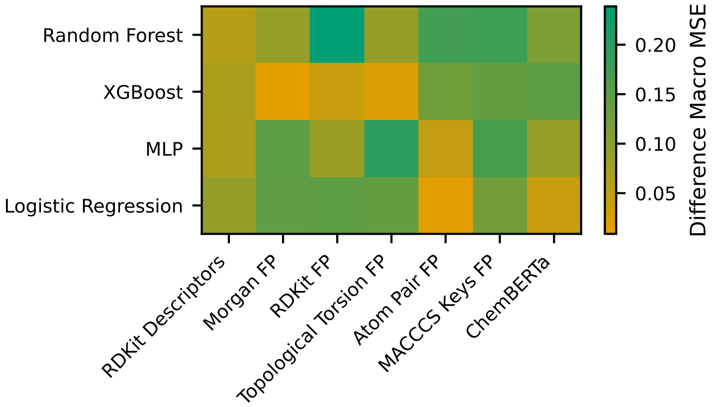

Figure S11 Differences in macro MSE between the indirect and direct approach models on the hold-out test set (direct subtracted from indirect). For each combination of molecular encoder (bottom) and predictor (left), the macro MSE of the direct model was subtracted from the indirect model of the same model combinations. MLP is multi-layer-perceptron and FP fingerprint. The difference regarding the message passing neural network using ChemProp[27,28] was 0.13, and the foundational model CheMeleon[29] 0.09.

S4 Direct ensemble model validation

Here, we provide Figure S12, which shows normed violin plots of experimental intensity ratings in comparison to our direct ensemble model predictions of the same molecules. It demonstrates the performance of our model on an independent hold-out test set of intensity ratings from Keller *et al.*[30] at a range of dilutions from 10^{-3} to 10^{-7}. The trend is similar to the lower dilution level in the main part: an increase in predicted odor strength correlates with a higher odor intensity rating. In addition, the plot shows a limitation of the model at low dilution (10^{-1}), where no trend was observed.

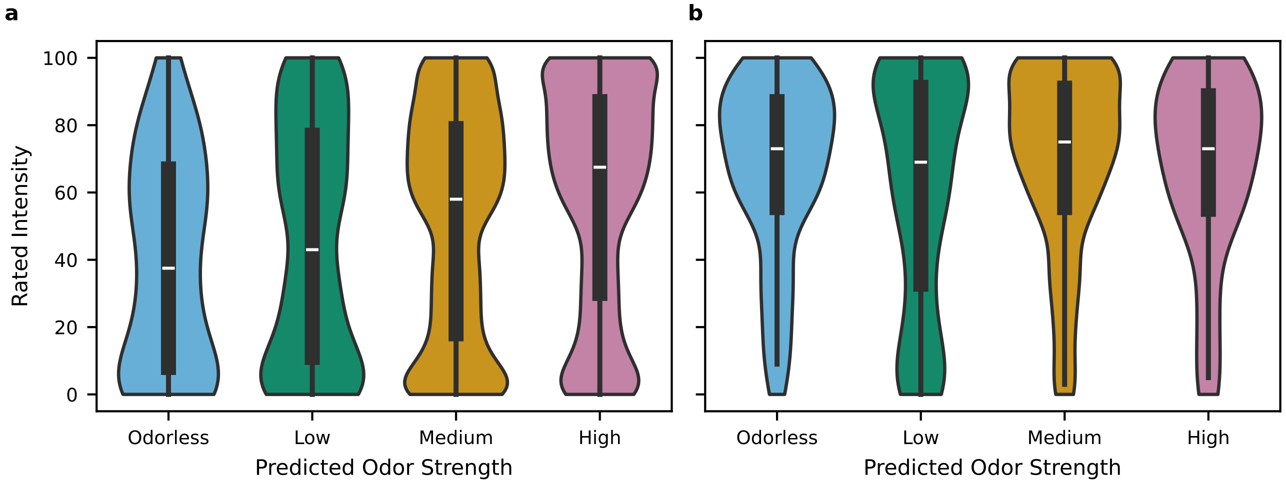

Figure S12 Area normed violin plots of the direct ensemble model predictions of the novel molecules from Keller *et al.*[30] and their rated intensities (from 0 to 100) at a) 10^{-3}, 10^{-5} and 10^{-7} and b) 10^{-1} dilution.

S5 SHAP feature importance analysis

We wanted to investigate feature importance of our direct ensemble model (RDKit Descriptors with MLP, Random Forest and XGB). However, we identified several correlated features as shown in Figure S13. Consequently, we grouped highly correlated features (correlation threshold 0.75) via agglomerative clustering. A correlation threshold of 0.75 was chosen due to a maximum of the silhouette score[6] (0.33) at this threshold. The silhouette scores for a range of thresholds are shown in Figure S14. The SHAP (SHapley Additive exPlanations) values on the test set (train set as background) of each feature of the five most influential groups are shown in Figure S15 (Weight and Shape), Figure S16 (Polarity), Figure S17 (Nitrogen-Polarity), Figure S18 (Alcohol Groups) and Figure S19 (Morgan Fingerprint Density). Figure S20 shows the SHAP feature importance of these groups per predicted odor strengths.

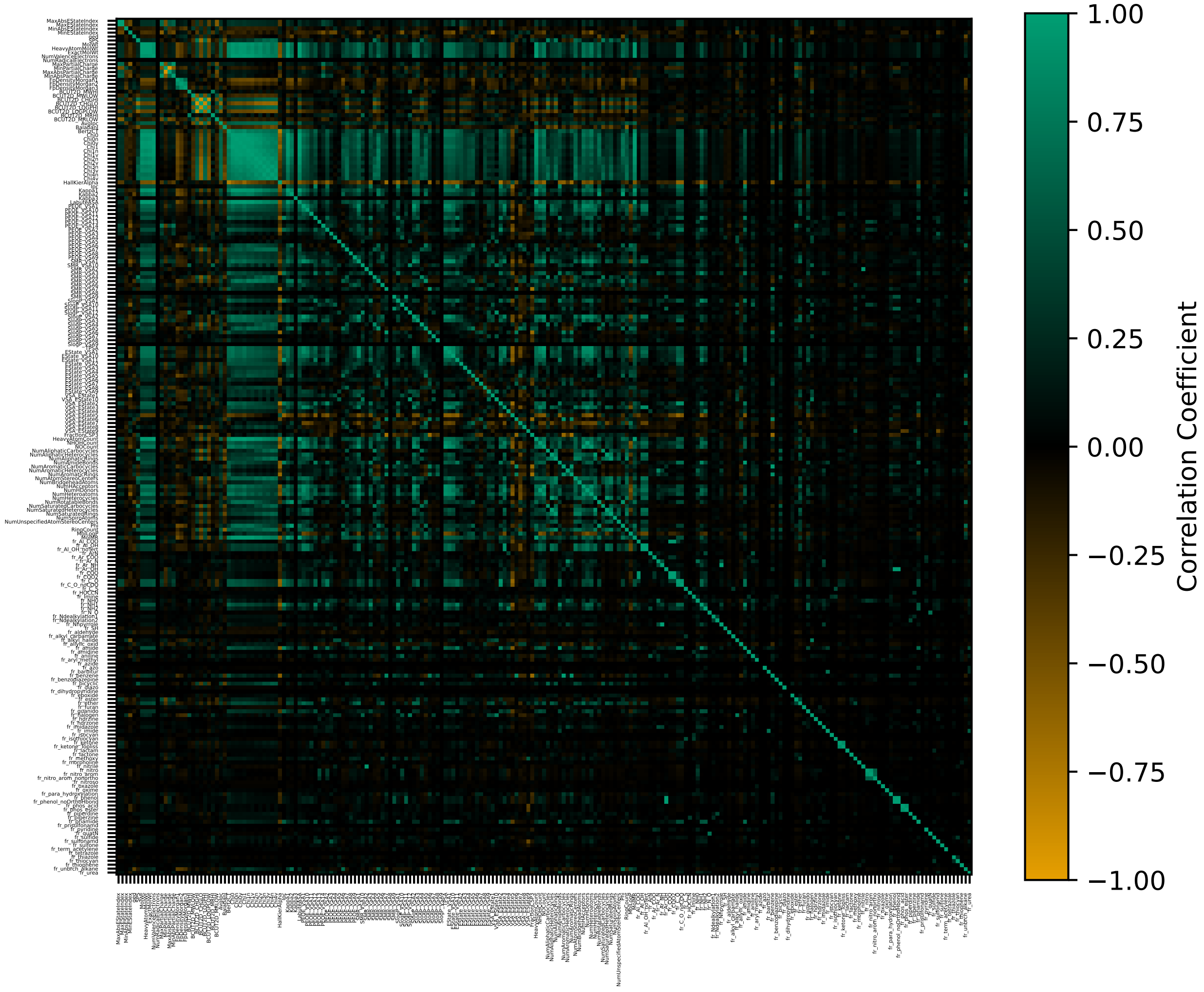

Figure S13 Correlation matrix of the 217 RDKit Descriptors of the molecules of our data set.

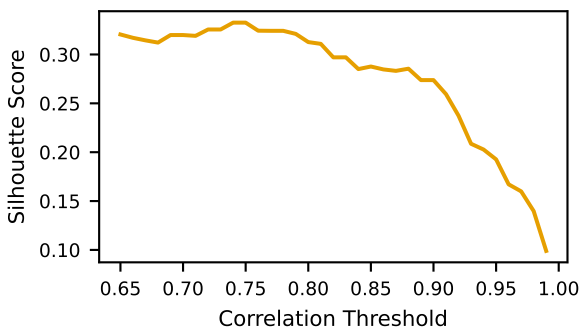

Figure S14 Silhouette scores for a range of correlation thresholds to cluster via agglomerative clustering.

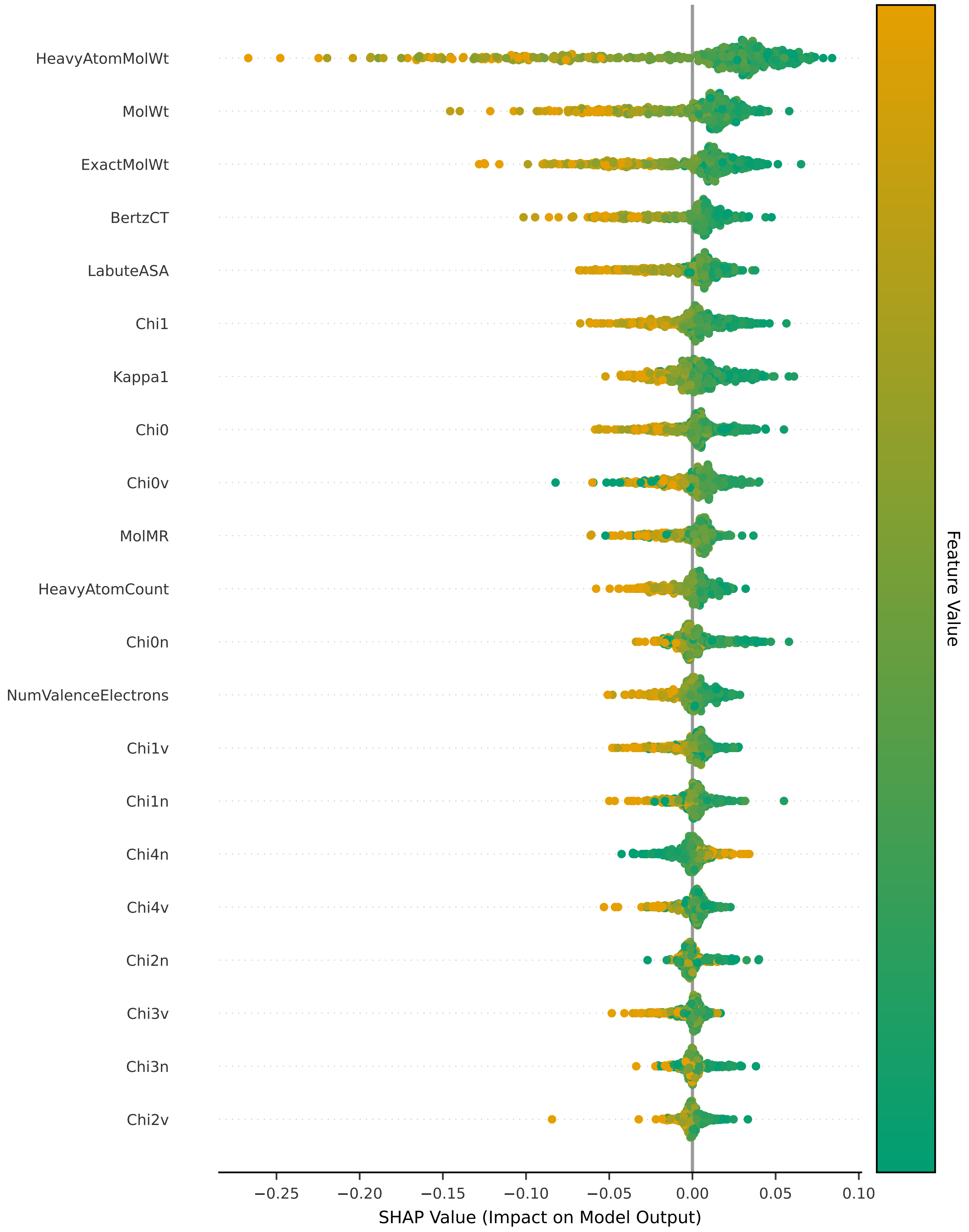

Figure S15 SHAP feature importance of the features of the 'Weight and Shape' group colored by the relative feature value on the test set using the train set as background. Each dot is an instance of the test set.

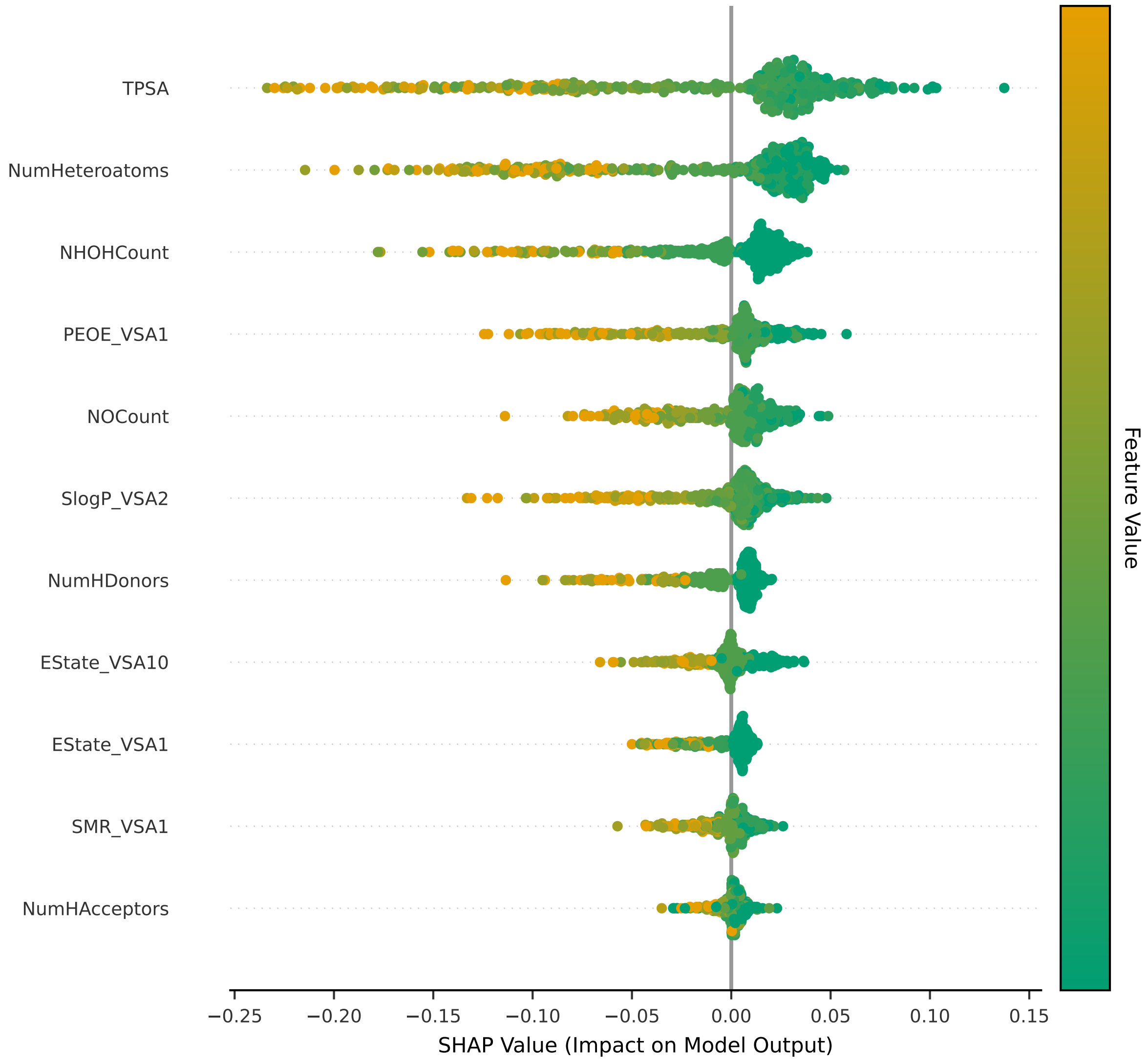

Figure S16 SHAP feature importance of the features of the 'Polarity' group colored by the relative feature value on the test set using the train set as background. Each dot is an instance of the test set.

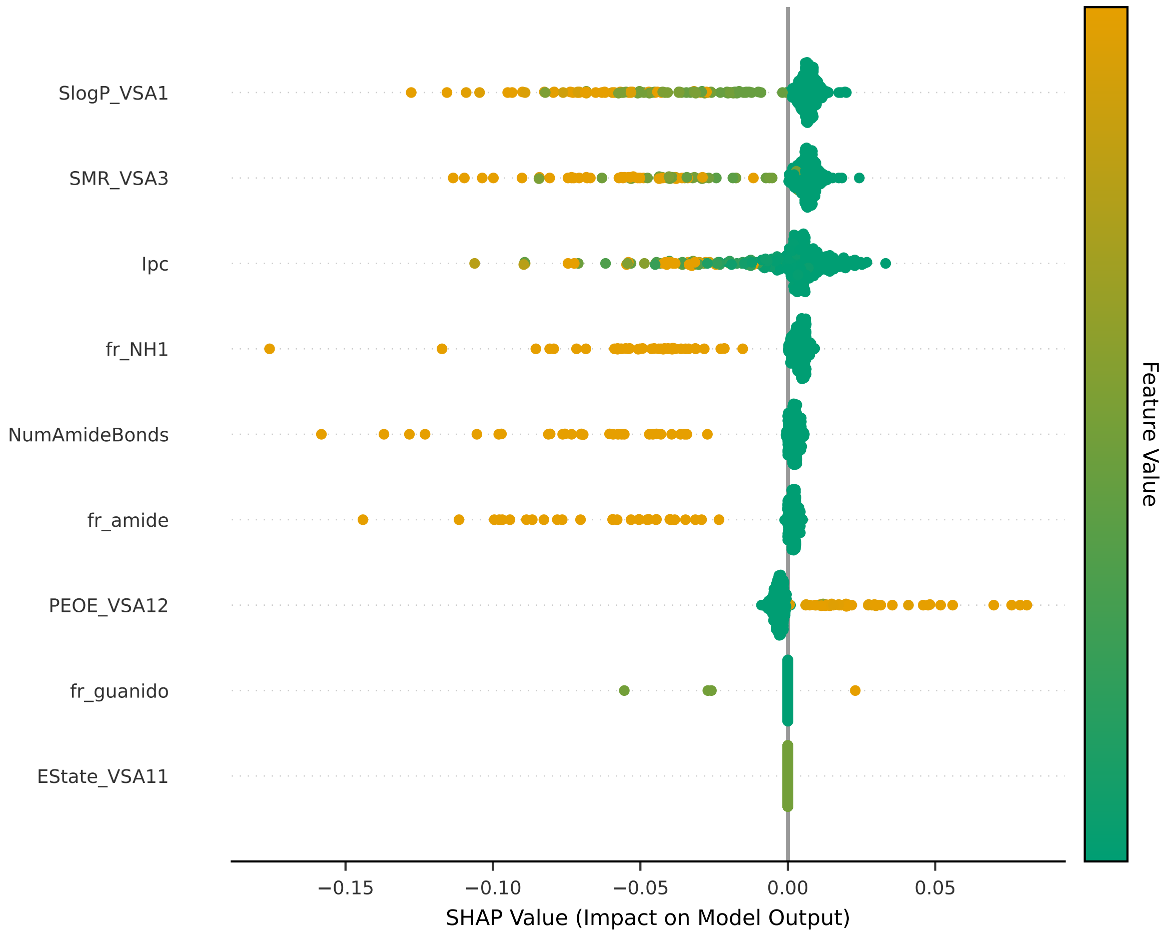

Figure S17 SHAP feature importance of the features of the 'Nitrogen-Polarity' group colored by the relative feature value on the test set using the train set as background. Each dot is an instance of the test set.

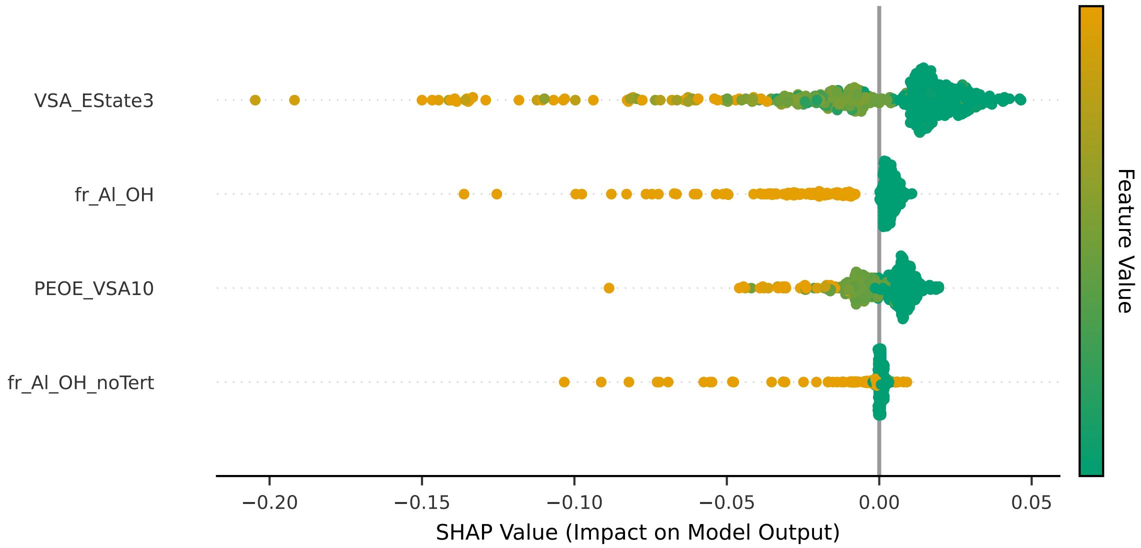

Figure S18 SHAP feature importance of the features of the 'Alcohol Groups' group colored by the relative feature value on the test set using the train set as background. Each dot is an instance of the test set.

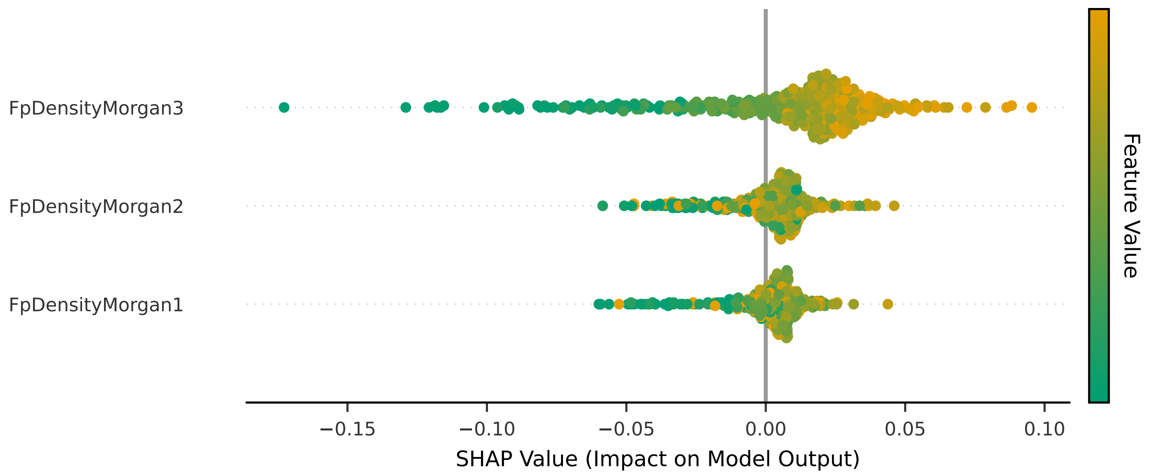

Figure S19 SHAP feature importance of the features of the 'Morgan Fingerprint Density' group colored by the relative feature value on the test set using the train set as background. Each dot is an instance of the test set.

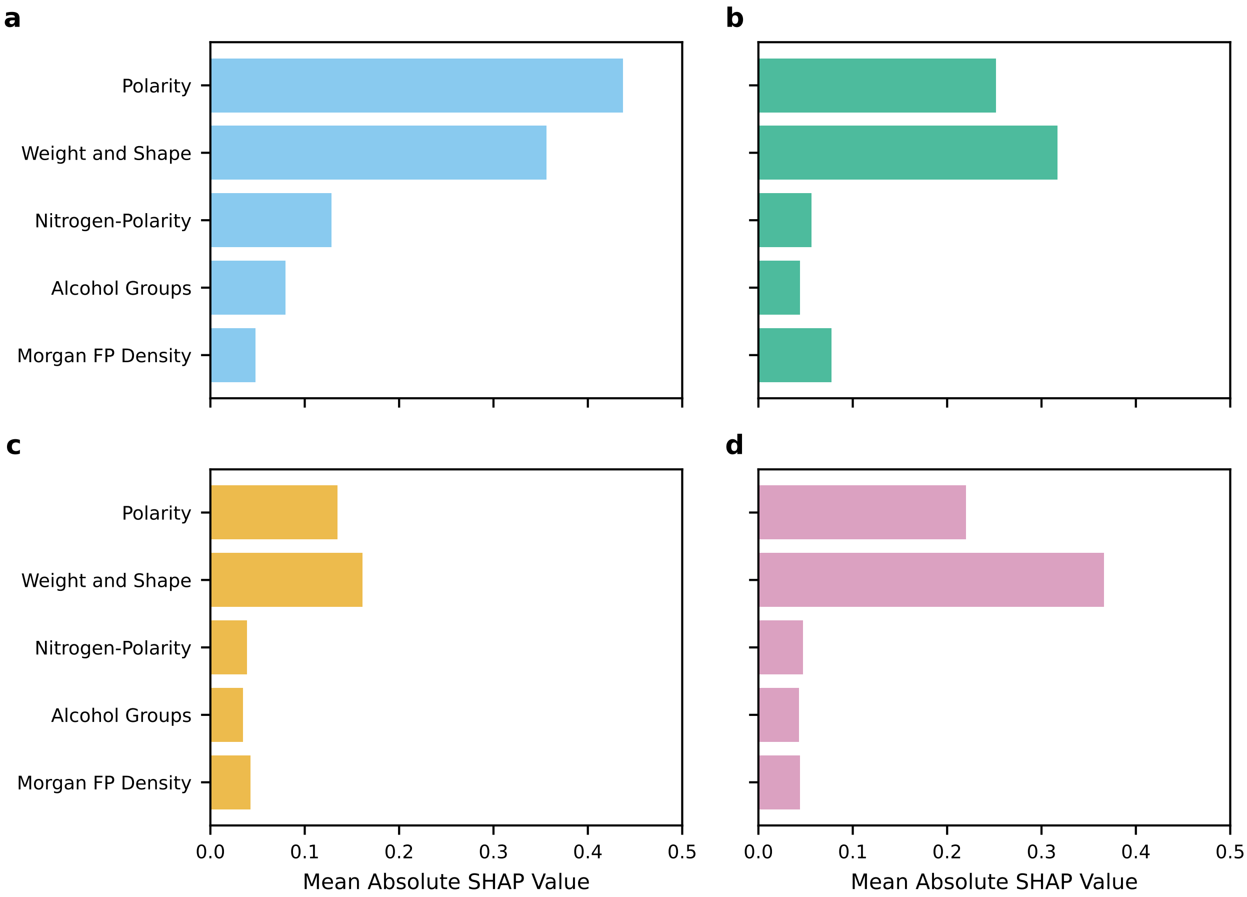

Figure S20 SHAP feature importance per odor strength prediction category: (a) odorless, (b) low, (c) medium and (d) high odor strength.

Local SHAP feature group contributions for four representative molecules are shown in Figure S21 to illustrate local interpretations. Smaller molecules, such as Ethylene Glycol or Indole, tend to shift the prediction toward higher odor strength, whereas larger molecules, such as Galaxolide, shift it toward lower odor strength. Furthermore, the prediction for the highly polar ethylene glycol is driven by the negative contributions of its alcohol groups and overall polarity, while the lower but sufficient polarity of Galaxolide and Indole contributes positively to their higher odor strengths.

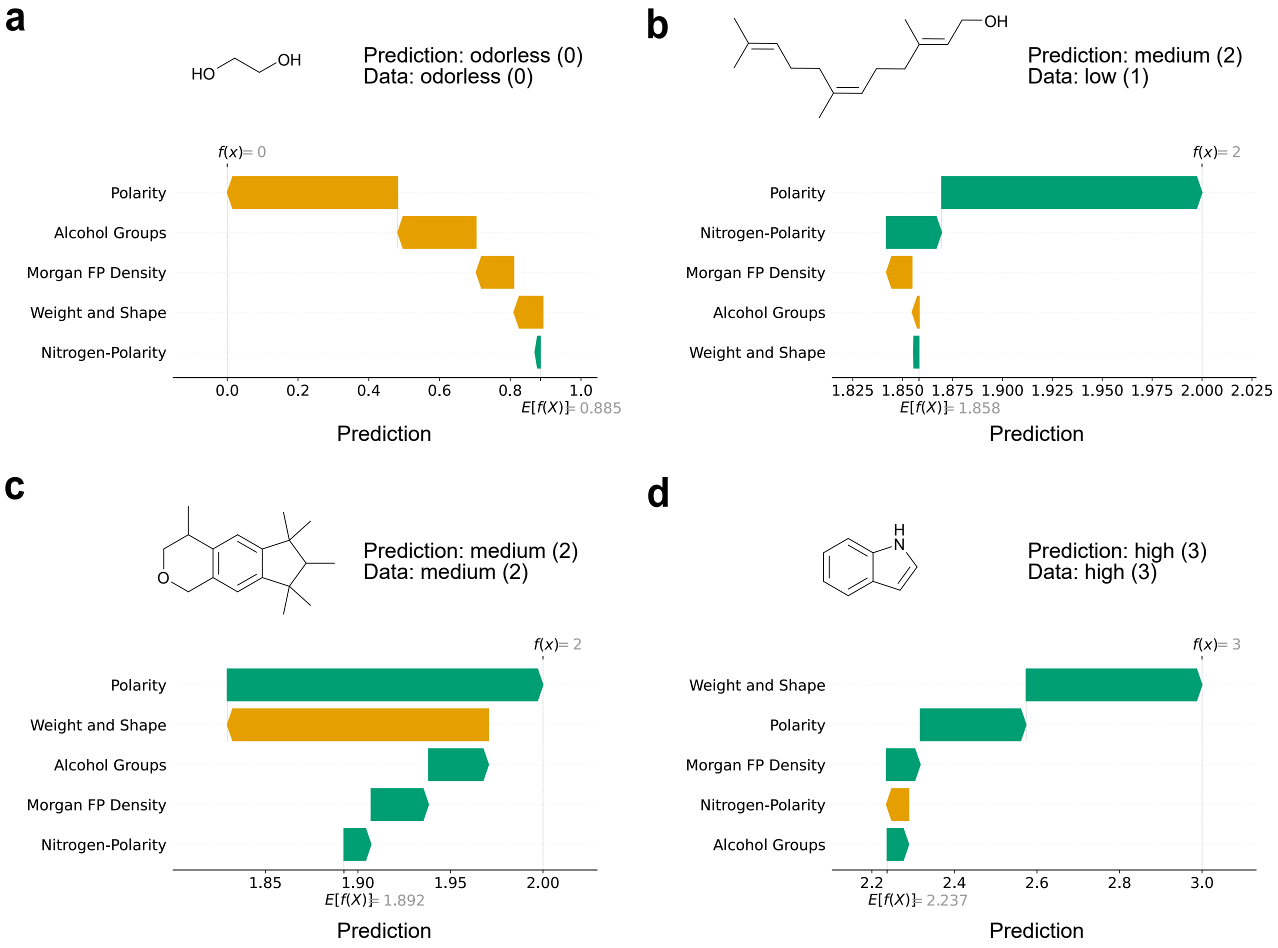

Figure S21 SHAP waterfall plots of the global most important feature groups for 4 example molecules of the data set using the train set as background. The contribution of each feature (SHAP value) to the prediction to deviate from the average background is shown. The molecules are (a) Ethylene glycol (odorless), (b) *E,Z*-Farnesol (low odor strength), (c) 1,3,4,6,7,8-Hexahydro-4,6,6,7,8,8-hexamethylcyclopenta[g]-2-benzopyran, also known as Galaxolide (medium odor strength), (d) Indole (high odor strength).

S6 Further Method Details

The following keywords were used to map a PubChem description to a corresponding odor strength:

- **Odor strength: description**
- odorless: odourless, odorless, no odour, no odor, very faint, very weak, very mild
- low: faint, weak
- high: very strong, very intense, very powerful, very pungent, very aromatic

The following 13 molecules in Table S20 were originally labeled as 'very high' odor strength and reclassified to 'high' odor strength.

Name	CAS	Canonical SMILES
ortho-thioguaiacol	7217-59-6	COc1ccccc1S
(Z)-6-nonenal	2277-19-2	CC/C=C\CCCCC=O
skatole	83-34-1	Cc1c[nH]c2ccccc12
isopropyl mercaptan	75-33-2	CC(C)S
caramel furanone	28664-35-9	CC1=C(O)C(=O)OC1C
2-mercaptopropionic acid	79-42-5	CC(S)C(=O)O
cortex pyridine	2110-18-1	c1ccc(CCCc2ccccn2)cc1
(Z)-4-heptenal	6728-31-0	CC/C=C\CCC=O
2-acetyl pyrazine	22047-25-2	CC(=O)c1cnccn1
2-acetyl thiazole	24295-03-2	CC(=O)c1nccs1
propyl mercaptan	107-03-9	CCCS
maple furanone	698-10-2	CCC1OC(=O)C(O)=C1C
Allyl Isothiocyanate		C=CCN=C=S

Table S20 Molecules which were originally labeled as 'very high' odor strength and reclassified to 'high' odor strength.

Notes and references

1 E. J. Mayhew, C. J. Arayata, R. C. Gerkin, B. K. Lee, J. M. Magill, L. L. Snyder, K. A. Little, C. W. Yu and J. D. Mainland, *Proc. Natl. Acad. Sci. U.S.A.*, 2022, **119**, e2116576119.
2 L. Ruddigkeit, R. van Deursen, L. C. Blum and J.-L. Reymond, *J. Chem. Inf. Model.*, 2012, **52**, 2864–2875.
3 L. Hubert and P. Arabie, *J. Classif*, 1985, **2**, 193–218.
4 A. Strehl and J. Ghosh, *J. Mach. Learn. Res.*, 2003, **3**, 583–617.
5 X. V. Nguyen, J. Epps and J. Bailey, *J. Mach. Learn. Res.*, 2010, **11**, 2837–2854.
6 P. J. Rousseeuw, *J. Comput. Appl. Math.*, 1987, **20**, 53–65.
7 S. Lloyd, *IEEE Trans. Inf. Theory*, 1982, **28**, 129–136.
8 D. J. Hand, G. J. McLachlan and K. E. Basford, *J. R. Stat. Soc. C: Appl. Stat.*, 1989, **38**, 384.
9 M. Ester, H.-P. Kriegel, J. Sander and X. Xu, *Data Min. Knowl. Discov.*, 1996, 226–231.
10 Jianbo Shi and J. Malik, *IEEE Trans. Pattern Anal. Mach. Intell.*, 2000, **22**, 888–905.
11 A. Ng, M. Jordan and Y. Weiss, *NIPS'01: Proceedings of the 15th International Conference on Neural Information Processing Systems: Natural and Synthetic*, MIT Press, Vancouver, British Columbia, Canada, 2001, vol. 14, pp. 849–856.
12 J. H. Ward, *J. Am. Stat. Assoc.*, 1963, **58**, 236–244.
13 T. Akiba, S. Sano, T. Yanase, T. Ohta and M. Koyama, *KDD '19: Proceedings of the 25th ACM SIGKDD International Conference on Knowledge Discovery & Data Mining*, Association for Computing Machinery, Anchorage, AK, USA, 2019, pp. 2623–2631.
14 H. L. Morgan, *J. Chem. Doc.*, 1965, **5**, 107–113.
15 D. Rogers and M. Hahn, *J. Chem. Inf. Model.*, 2010, **50**, 742–754.
16 *RDKit: Open-source Cheminformatics (Release_2025.03.5)*, https://zenodo.org/doi/10.5281/zenodo.591637, (accessed October 2025).
17 R. Nilakantan, N. Bauman, J. S. Dixon and R. Venkataraghavan, *J. Chem. Inf. Comput. Sci.*, 1987, **27**, 82–85.
18 R. E. Carhart, D. H. Smith and R. Venkataraghavan, *J. Chem. Inf. Comput. Sci.*, 1985, **25**, 64–73.
19 W. Ahmad, E. Simon, S. Chithrananda, G. Grand and B. Ramsundar, *arXiv*, 2022, preprint, arXiv:2209.01712, https://arxiv.org/abs/2209.01712.
20 J. L. Durant, B. A. Leland, D. R. Henry and J. G. Nourse, *J. Chem. Inf. Comput. Sci.*, 2002, **42**, 1273–1280.
21 P.-F. Verhulst, *Nouv. Mem. Acad. R. Sci. Bruxelles*, 1845, **18**, 1–38.

22 L. Breiman, *Mach. Learn.*, 2001, **45**, 5–32.

23 T. Chen and C. Guestrin, *KDD '16: Proceedings of the 22nd ACM SIGKDD International Conference on Knowledge Discovery and Data Mining*, Association for Computing Machinery, San Francisco, California, USA, 2016, pp. 785–794.

24 D. E. Rumelhart, G. E. Hinton and R. J. Williams, *Nature*, 1986, **323**, 533–536.

25 P. Adam, A. Paszke, G. Sam, S. Gross, M. Francisco, F. Massa, A. Lerer, B. James, J. T. Bradbury, C. Gregory, G. Chanan, K. Trevor, T. Killeen, L. Zeming, Z. Lin, G. Natalia, N. Gimelshein, A. Luca, L. Antiga, D. Alban, A. Desmaison, K. Andreas, A. Köpf, Y. Edward, E. Yang, D. Zach, Z. DeVito, R. Martin, M. Raison, T. Alykhan, A. Tejani, C. Sasank, S. Chilamkurthy, S. Benoit, B. Steiner, F. Lu, F. Liu, B. Junjie, J. Bai, C. Soumith and S. Chintala, *arXiv*, 2019, preprint, arXiv:1912.01703, https://arxiv.org/abs/1912.01703.

26 W. Cao, V. Mirjalili and S. Raschka, *Pattern Recognit. Lett.*, 2020, **140**, 325–331.

27 K. Yang, K. Swanson, W. Jin, C. W. Coley, P. Eiden, H. Gao, A. Guzmán-Pérez, A. Guzman-Perez, T. Hopper, B. Kelley, M. Mathea, A. Palmer, V. Settels, T. S. Jaakkola, K. F. Jensen and R. Barzilay, *J. Chem. Inf. Model.*, 2019, **59**, 3370–3388.

28 E. Heid, K. P. Greenman, Y. Chung, S.-C. Li, D. E. Graff, F. H. Vermeire, H. Wu, W. H. Green and C. J. McGill, *J. Chem. Inf. Model.*, 2024, **64**, 9–17.

29 J. Burns, A. S. Zalte and W. Green, *arXiv*, 2025, preprint, arXiv: 2506.15792, https://arxiv.org/abs/2506.15792.

30 A. Keller, R. C. Gerkin, Y. Guan, A. Dhurandhar, G. Turu, B. Szalai, J. D. Mainland, Y. Ihara, C. W. Yu, R. Wolfinger, C. Vens, L. Schietgat, K. De Grave, R. Norel, D. O. P. Consortium, G. Stolovitzky, G. A. Cecchi, L. B. Vosshall and P. Meyer, *Science*, 2017, **355**, 820–826.
